# Bioactive Compounds of Marine Algae and Their Potential Health and Nutraceutical Applications: A Review

**DOI:** 10.3390/md23040152

**Published:** 2025-03-31

**Authors:** Emin Cadar, Antoanela Popescu, Ana-Maria-Laura Dragan, Ana-Maria Pesterau, Carolina Pascale, Valentina Anuta, Irina Prasacu, Bruno Stefan Velescu, Cezar Laurentiu Tomescu, Claudia Florina Bogdan-Andreescu, Rodica Sirbu, Ana-Maria Ionescu

**Affiliations:** 1 Faculty of Pharmacy, “Ovidius” University of Constanta, Capitan Aviator Al. Serbanescu Street, No. 6, Campus, Corp C, 900470 Constanta, Romania; emin.cadar@gmail.com (E.C.); antoniapopescu2002@yahoo.co.uk (A.P.); 2 Organizing Institution for Doctoral University Studies of “Carol Davila”, University of Medicine and Pharmacy of Bucharest, Dionisie Lupu Street, No. 37, Sector 2, 020021 Bucharest, Romania; ana-maria.pesterau@drd.umfcd.ro (A.-M.P.); carolina.pascale@drd.umfcd.ro (C.P.); 3 Faculty of Pharmacy, “Carol Davila” University of Medicine and Pharmacy of Bucharest, Traian Vuia Street, No. 6, Sector 2, 020021 Bucharest, Romania; valentina.anuta@umfcd.ro (V.A.); irina.prasacu@umfcd.ro (I.P.); bruno_velescu@yahoo.co.uk (B.S.V.); 4 Faculty of Medicine, “Ovidius” University of Constanta, University Alley, No. 1, Campus, Corp B, 900470 Constanta, Romania; tomescu.cezar.laurentiu@gmail.com (C.L.T.); anaiulius@yahoo.com (A.-M.I.); 5 “Sf. Ap. Andrei” County Clinical Emergency Hospital, Tomis Bvd., No. 145, 900591 Constanta, Romania; 6 Faculty of Dental Medicine, Department of Speciality Disciplines, “Titu Maiorescu” University, 031593 Bucharest, Romania; claudia.andreescu@prof.utm.ro; 7 Clinical Hospital C F Constanta, 1 Mai Bvd., No. 3–5, 900123 Constanta, Romania

**Keywords:** seaweeds, nutritional composition, antioxidant properties, anti-inflammatory effects, anti-cancer potential, functional foods, dietary supplements, nutraceuticals

## Abstract

Currently, marine algae are still an under-exploited natural bioresource of bioactive compounds. Seaweeds represent a sustainable source for obtaining bioactive compounds that can be useful for the fabrication of new active products with biomedical benefits and applications as biomedicinals and nutraceuticals. The objective of this review is to highlight scientific papers that identify biocompounds from marine macroalgae and emphasize their benefits. The method used was data analysis to systematize information to identify biocompounds and their various benefits in pharmaceuticals, cosmetics, and nutraceuticals. The research results demonstrate the multiple uses of seaweeds. As pharmaceuticals, seaweeds are rich sources of bioactive compounds like polysaccharides, protein compounds, pigments, and polyphenols, which have demonstrated various pharmacological activities such as antioxidant, antibacterial, anti-inflammatory, antiviral, anticoagulant, and potentially anticarcinogenic effects. Seaweed has gained recognition as a functional food and offers a unique set of compounds that promote body health, including vitamins, minerals, and antioxidants. In conclusion, the importance of this review is to expand the possibilities for utilizing natural resources by broadening the areas of research for human health and marine nutraceuticals.

## 1. Introduction

Maintaining health is humanity’s most important concern. The aquatic environment is one of the abundant sources of bioactive substances that are proven to be good for human health. The biodiversity of the marine ecosystem provides a large reservoir of novel bioactive nutrients for both marine organisms and humans [1]. In this regard, seaweeds represent one of the potential sources of marine bioactive compounds [1,2]. Seaweeds, as aquatic plants, are eukaryotic, photosynthetic organisms found in both marine and freshwater [3]. Seaweeds have been consumed as food since prehistoric times in the Chinese, Japanese, and Korean diets, with written evidence attesting to their use. Although the consumption of seaweeds by humans dates back to ancient times, the study of bioactive compounds started to develop significantly in recent decades, as shown by Cadar et al. (2019) [4]. In Europe, seaweed consumption has increased with people’s interest in the use of natural marine products and functional foods as shown by Ferrara et al. (2020) and Ferdouse et al. (2018) [5,6]. The use of macroalgae as food and feed has been addressed by Embling et al. (2022) and Cherry et al. (2019) [7,8]. Cadar et al. (2019) showed that seaweeds can be utilized for their ability to accumulate heavy metals which are considered as pollutants to the marine environment [9]. Seaweeds can also be used as an ingredient in combination with other marine-derived materials to obtain preparations usable in the treatment of some dermal diseases as shown by Cherim et. al. (2019) and Sirbu et al. (2019) [10,11]. The biggest use of macroalgae is as food for humans and animals. Araújo et al. (2021) indicated that although algae production from the marine environment accounts for 68% of the total, in Europe, algae culture (32% of the total) has started to expand [12]. Cai et al. (2021) analyzed the Global Status of Algae Production in FAO from 1990 to 2019 and showed that the world production of brown and red algae from aquaculture has increased, but the cultivation of green algae has decreased by about half [13]. Worldwide, according to the global trade statistics reported to FAO on world seaweed production, Asia contributed 97.38%, the two Americas (North and South) 1.36%, Europe 0.8%, followed by Africa 0.41% and Oceania 0.05% [13,14]. The development of macroalgae aquaculture has evolved by optimizing culture systems for several algal species. Seaweed can be a source of secondary metabolites that have multiple beneficial health effects for different diseases, as reported by André et al. (2021) [15]. Ouyang et al. (2021) reported that algal polysaccharides as nutraceuticals present an important potential for combating cancer [16]. Activities of marine algae in anti-HPV and anti-cervical cancer are studied by Moga et al. (2021) [17]. The properties of the fucoidan extracted from brown seaweeds, such as anti-inflammatory, immunomodulatory, antitumoral, anticoagulant, neuroprotective antioxidant, and cardioprotective, were studied by Saeed et al. (2021) [18]. Lomartire et al. (2021) presented the biocompounds of macroalgae with a polysaccharide structure and their applications for medical benefits [19]. In recent years, the demand for nutritious and nourishing food products has been steadily increasing. According to the FAO analysis from 2022, this global growth for healthy food is accounted for by the development of the aquaculture sector, which grew worldwide in 2019 by approximately 30% compared to the previous period [20]. Marine macroalgae constitute a rich resource of bioactive compounds such as polysaccharides, polyphenols, minerals, vitamins, carotenoids, fibers, proteins, amino acids, and polyunsaturated fatty acids, which are important for health as medicines or nutraceutical compounds, as shown by Park et al. (2023), Beaumont et al. (2021), Caf et al. (2019), and [21,22,23].

Seaweed industrialization has progressed significantly, with applications extending beyond traditional food consumption into high-value sectors such as cosmetics, biomedicine, and biofuels. Innovations in farming techniques, including large-scale mariculture and controlled bioreactor systems, have increased production efficiency and sustainability. Advances in biotechnology, including genetic modification and optimized extraction methods, further contribute to maximizing the potential of compounds derived from seaweed. Despite these advances, challenges such as environmental issues and resource management remain areas of active research. In addition to industrial progress, political support and areas of legislative regulation play a crucial role in shaping the future of seaweed utilization. Governments around the world are implementing policies to promote sustainable aquaculture, stimulate research, and regulate the marketing of algae products. The European Union, for example, has introduced directives to encourage sustainable seaweed farming, while the United States and Asian countries have included seaweed in their blue economy strategies.

These policy-driven initiatives help to bridge the gap between scientific discoveries and commercial applications, ensuring that seaweed remains a viable resource for future innovation.

The aim of our study is to analyze the data on biocompounds with significant biological activity that have pharmaceutical, biomedical, and nutraceutical benefits. Methods of the extraction and the structures of active biocompounds such as polysaccharides, terpenoid compounds, proteins, saturated and unsaturated fatty acids, and polyphenolic and flavonoid compounds and data on pigments, vitamins, and minerals existing in marine macroalgae are described. The biomedical and nutraceutical applications due to these biocompounds are systematized. The biomedical and nutraceutical benefits are presented based on the biological activities of secondary metabolites in macroalgae.

## 2. Methodology

The research methodology involved the systematic collection and analysis of literature data from 2017 to 2024. The methodology for selecting the reviewed papers followed a systematic approach to ensure the inclusion of high-quality and relevant studies on bioactive compounds from marine algae. The selection process involved a structured literature search using multiple scientific databases, including ScienceDirect, SCOPUS, Google Scholar, and Web of Science. The search strategy was designed to retrieve the most relevant articles by using a combination of carefully selected keywords and Boolean operators. Keywords included the following: “algal bioactive principles”, “marine macroalgae”, “algae primary and secondary metabolites”, “algal pharmaceuticals”, “seaweed-based medicines”, “seaweed health benefits”, “anti-inflammatory potential”, “antioxidant properties”, “pharmaceutical compounds from marine algae”, “biomedical applications”, “marine-derived drugs”, “seaweed supplements”, “nutraceutical compounds from marine algae”, “seaweed skincare”, and “algal cosmeceuticals”. These keywords were applied in different combinations to maximize the retrieval of relevant literature.

For inclusion, the studies had to meet specific criteria: (1) published between 2017 and 2024 to ensure up-to-date information, (2) peer-reviewed articles focusing on bioactive compounds from marine macroalgae and their biomedical, pharmaceutical, and nutraceutical applications, (3) studies providing detailed information on the extraction, characterization, and functional properties of these compounds, and (4) research presenting experimental or clinical evidence on the efficacy and safety of bioactive compounds derived from marine algae.

Exclusion criteria were also applied to eliminate irrelevant or low-quality studies. Articles were excluded if they (1) focused primarily on freshwater algae rather than marine macroalgae, (2) lacked experimental data and were purely theoretical or speculative, (3) were duplicate studies or conference abstracts without full research findings, (4) did not provide substantial information on bioactive compound applications, and (5) were published in predatory or non-peer-reviewed journals.

The final selection of studies was conducted by screening titles and abstracts for relevance, followed by a full-text review of shortlisted papers. Any discrepancies in selection were resolved through discussion among the researchers to ensure objectivity and comprehensiveness in the review. This rigorous methodology ensured a high-quality synthesis of relevant literature on the bioactive potential of marine algae.

## 3. Chemical Bioactive Compounds from Macroalgae

### 3.1. Biodiversity of Algae

Macroalgae are classified into three main taxa: Chlorophyta (green algae), Rhodophyta (red algae), and Phaeophyceae (brown algae), based on factors such as photosynthetic pigment type, cell wall composition, flagella type, and storage compounds as shown by Zhong et al. (2020), Pereira (2021), and Cadar et al. (2019) [24,25,26]. In marine environments, the presence of seaweeds significantly influences aquatic ecosystems, with various factors affecting their biochemical composition. Macroalgae are commonly found in abundance on rocky coastal shores, and some species form extensive underwater forests, known as algae forests, which can cover over 50 m² as shown by Pereira (2021) and Gaspar et al. (2020) [25,27]. According to Veluchamy, C. et al. (2020) and Kennedy, J. (2019), approximately 7000 species of red algae, distinguished by their bright coloration due to phycoerythrin pigment, and 4000 species of green algae, which owe their color to chlorophyll pigments, have been identified so far [28,29]. Green algae inhabit marine and freshwater environments as well as wet soils [30,31]. Sirbu et al. (2019) and Tanna et al. (2021) showed that *Ulva lactuca* (Chlorophyta) is commonly found in coastal marine waters and tidal pools [31,32]. Tanna et al. (2021) showed that red algae, which are typically multicellular and have a reddish hue, thrive at greater depths than green and brown algae because they efficiently absorb blue light [32]. Hakim et al. showed that brown algae are abundant on rocky shores in temperate regions [33]. Seaweeds endure harsh environmental conditions influenced by various external factors, including climatic fluctuations, seasonal and temperature changes, geographic location, mineral concentration, pH levels, light availability, and contaminants in the aquatic environment. These factors can significantly impact their biochemical composition. Badar et al. (2021) noted that macroalgae can thrive in challenging conditions with high heavy metal concentrations and can be utilized as a source of energy, biochemical components, and food [34]. Their ability to adapt to diverse marine environments accounts for the wide variability in their secondary metabolites and biochemical composition.

### 3.2. Seaweed Cultivation and Harvesting

With the rising global demand for natural food, mariculture has become essential for sustainably supplying functional foods and medical products derived from marine resources. Algae farming is now practiced in over 30 countries, utilizing cold, tropical, and temperate waters for food, pharmaceuticals, and biofuel production. According to Buschmann et al. (2019), annual seaweed harvests exceed 2 million tons in countries such as China, Japan, South Korea, Indonesia, and France [35]. In South American countries such as Chile, algae are harvested for their medicinal benefits, thus Cochayuyo (*Durvillaea incurvata*) (Phaeophyceae) extracts have been tested for potential uses as an antioxidant-active ingredient as reported by Pacheco et al. (2023) and for the potential to fight age-related diseases as reported by Muñoz-Molina, et al. (2024) [36,37]. Macroalgae cultivation has become increasingly profitable, as it requires no freshwater and can be sustained year-round in both coastal and offshore areas through various methods. Kumar et al. (2021) noted that cultivation techniques depend on location and seaweed species [38]. Globally, species such as *Gracilaria* sp., *Eucheuma* sp., *Laminaria* sp., *Kappaphycus* sp., *Gelidium* sp., *Pyropia* sp., *Undaria* sp., *Saccharina* sp., *Sargassum* sp., and *Ulva* sp. are widely farmed [38]. Suthar et al. (2019) emphasized that achieving high algal biomass requires careful monitoring of environmental factors, including temperature, light, salinity, nutrient availability, water movement, cultivation depth, herbivorous fish, and epiphytes in the marine ecosystem [39]. Different methods are used for seaweed cultivation depending on the location and species. Techniques such as rope, raft, tubular net, and photo-bioreactor systems have been developed for certain species of *Ulva* sp. Hwang et al. (2020) reviewed macroalgae cultivation and harvesting methods used in South Korea [40]. The challenges of genetic degradation, poor environmental adaptation, and increased disease incidence have led to the preference for farm-based seaweed cultivation, which ensures higher-quality yields.

Obando et al. (2022) demonstrated that optimizing macroalgae cultivation for growth control, development, and secondary metabolite production requires more advanced techniques. These include selecting competent cells for growth induction using chemical regulators in the culture medium, as well as incorporating carbon and nitrogen sources to support the growth of protoplast and algal cell cultures. Such approaches are essential for enhancing macroalgae cultivation to meet various objectives [41].

### 3.3. Biochemical Composition of Algae

The nutritional composition of seaweeds includes bioactive compounds with significant medical, nutraceutical, and food-related benefits. Selecting the appropriate algae for study is crucial to ensuring that the biomass is clean and sourced from designated areas, preventing contamination that could compromise its quality, whether obtained from natural environments or aquaculture systems. The biochemical composition of algae is influenced by various factors, including biotic factors, such as symbiotic relationships with other organisms, and abiotic factors, such as temperature, light, carbon source, and nutrient availability as shown by Regal et al. (2020), Olsson et al. (2020), and Alvarez-Gomez (2019) [42,43,44]. Another key challenge is the collection of algal biomass, as the lack of standardized collection methods can impact the entire extraction process. Several factors affect the composition of the algal extract and the bioactivity of its compounds, including biomass drying methods, extraction techniques, solvent type and ratio, working temperature, and extraction duration as shown by Sobuj et al. (2021), Mansur et al. (2020), Uribe et al. (2019), and Getachew et al. (2022) [45,46,47,48].

#### 3.3.1. The Extraction Methods of Biocompounds from Seaweeds

**Figure 1:**
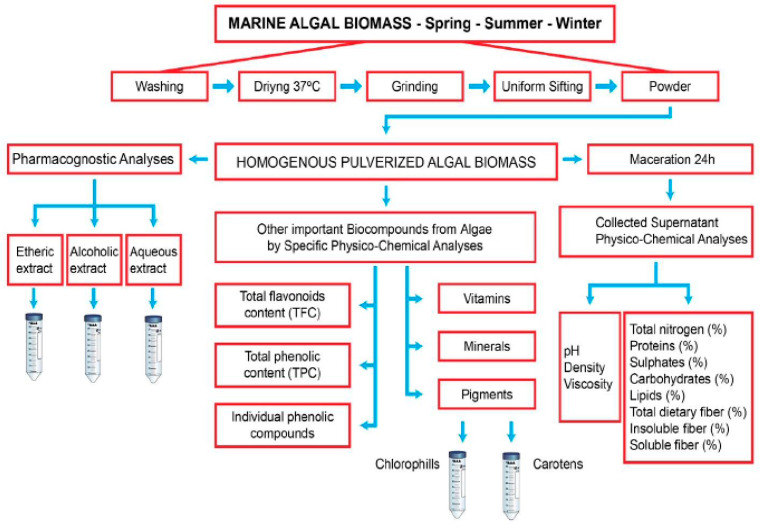
The scheme of the processes in physicochemical analysis of seaweed biocompounds.

The process of extracting biocompounds from seaweed involves multiple stages of physicochemical analysis, including pre-treatment, extraction, and purification. Figure 1 presents the pre-treatment processes, followed by the isolation and identification of bioactive compounds from macroalgae harvested from the Black Sea, as outlined by Cadar et al. (2023) [49]. The extraction of bioactive compounds from algae can be performed using classical methods, such as Conventional Chemical Extraction, which requires further purification and separation through chromatographic and spectrophotometric techniques (e.g., UV-VIS) or coupled methods like GC-MS to ensure extract purity.

**Figure 2:**
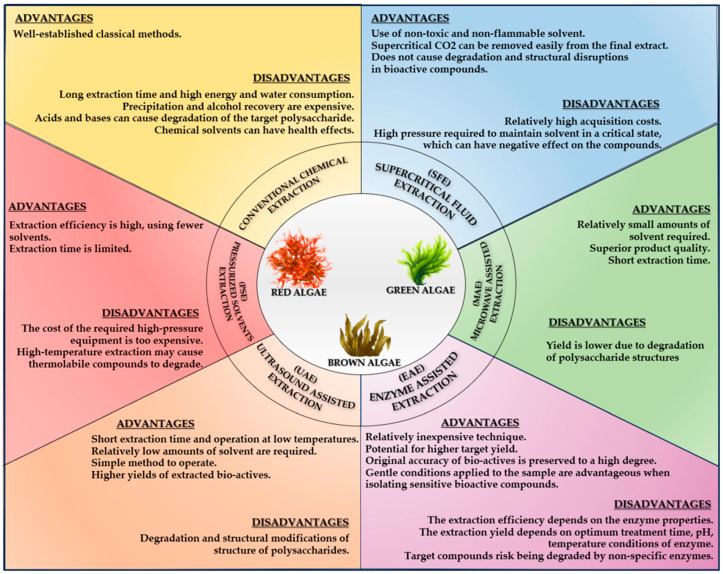
Different conventional and alternative extractive methods used to isolate biocompounds from seaweeds.

Conventional Solvent Extraction (CSE) encompasses techniques such as Soxhlet extraction, solid–liquid extraction (SLE), and liquid–liquid extraction (LLE). Additionally, water-based extraction methods (boiling, autoclaving, and halogenation) and acid or alkaline hydrolysis are commonly employed. Several modern alternatives to Conventional Chemical Extraction have been proposed by Amlani et al. (2022), and Jönsson et al. (2020). These include microwave-assisted extraction (MAE), enzyme-assisted extraction (EAE), supercritical fluid extraction (SFE), ultrasound-assisted extraction (UAE), and pressurized solvent extraction (PSE), which offer improved efficiency for isolating bioactive compounds [50,51]. It is necessary to perform the validation of the extraction and purification methods by using statistical validation methods with the establishment of validation parameters for each method as done by Sirbu et al. (2019) [52]. Figure 2 illustrates the various extraction methods along with their respective advantages and disadvantages for obtaining algal extracts.

#### 3.3.2. Proximate Nutritional Composition

The nutritional composition of algae is presented in the table below. Table 1 includes the following data: moisture and ash content, sulfate compound and total nitrogen content, and protein, lipid, carbohydrate and total dietary fiber content, which have been reported for algae that are representative of the three categories of algae: green, red, and brown. The green algae studied come from different marine habitats found in different seas and oceans. The green algae are 5 species of *Ulva* sp., 3 species of *Ulva* (formerly *Enteromorpha*), 2 species of *Cladophora vagabunda*, 1 species *Acrosiphonia orientalis*, and 3 species of *Caulerpa* sp. The Black Sea algae *Ulva lactuca*, *Ulva intestinalis* (formerly *Enteromorpha intestinalis*), and *Cladophora vagabunda* were reported by Sirbu et al. (2019) and Cadar et al. (2023) [31,49]. The Arabian Sea algae, *Ulva lactuca*, *Acrosiphonia orientalis*, and *Caulerpa scalpelliformis*, were reported by Choudhary et al. (2023) [53]. Algae from Atlantic water, *Ulva rigida* and *Caulerpa lentillifera*, were studied by Morais et al. (2020) [54]. The green algae from Indian waters, *Ulva lactuca* (formerly *Ulva fasciata*) and *Ulva flexuosa* (formerly *Enteromorpha flexuosa*), were studied by Ganesan et al. (2020) [55]. The green algae from the Gulf of Gökova of Aegean Sea, *Ulva intestinalis* (formerly *Enteromorpha intestinalis*), were investigated by Metin et al. (2018) [56]. All these results are systematized in Table 1. The red algae examined in this study come from various marine habitats. Cadar E. (2017) investigated marine algae from the Black Sea, specifically *Ceranium virgatum* (formerly *Ceramium rubrum*) (Rhodophyta) [57].

Choudhary et al. (2023) studied *Scinaia carnosa* and *Halymenia porphyriformis* from the Arabian Sea [53]. Morais et al. (2020) analyzed *Palmaria palmata* and *Porphyra umbilicalis* from Atlantic waters [54]. Ganesan et al. (2020) researched *Acanthophora spicifera* and *Gracilaria edulis* from Indian waters [55]. Premarathna et al. (2022) reported on *Jania pedunculata* var. *adhaerens* (formerly *Jania adhaerens*) and *Gracilaria corticata* from the coastal waters of Sri Lanka [58]. Rosemary et al. (2019) provided data on *Gracilaria corticata* and *Gracilaria edulis* from the southeastern coast of India [59]. Additionally, Farghl et al. (2021) published findings on *Laurencia obtusa* from the Red Sea coast [60]. Similarly, the brown algae considered in this study are also presented in Table 1. Choudhary et al. (2023) examined *Iyengaria stellata* and *Sargassum linearifolium* from Arabian Sea waters [53]. Morais et al. (2020) analyzed several brown algae species from Atlantic waters, including *Fucus vesiculosus*, *Laminaria digitata*, *Undaria pinnatifida*, and *Saccharina latissima* [54]. Ganesan et al. (2020) studied the nutritional composition of *Padina gymnospora* from Indian Ocean waters [55]. Cadar E. (2017) provided data on the nutritional composition of the brown alga *Gongolaria barbata* (formerly *Cystoseira barbata*) from Black Sea waters [57]. Premarathna et al. (2022) reported the biochemical composition of the brown algae *Sargassum ilicifolium* and *Sargassum polycystum* from the coastal waters of Sri Lanka [58]. Praiboon et al. (2018) examined the biochemical composition of *Sargassum oligocystum* from the Indo-West Pacific region [61]. Ilyas et al. (2023) studied the proximate composition of the brown alga *Himanthalia elongata* from the North-eastern Atlantic Ocean [62]. Fouda et al. (2019) analyzed the chemical composition of *Sargassum asperifolium* from the Red Sea, specifically the Hurghada Coast [63]. Carbohydrates are present in all types of algae, varying in proportion and structure. In green algae, polysaccharides with ulvan structures are predominant, along with other structural types. The carbohydrate content ranges from 62.37% in *Cladophora vagabunda* from the Black Sea, as reported by Cadar et al. (2023), to 16% in *Acrosiphonia orientalis* from the Arabian Sea, as documented by Choudhary et al. (2023) [49,53]. In red algae, carbohydrate content varies from 56% in *Palmaria palmata* from Atlantic waters, reported by Morais et al. (2020) [54], to as low as 4.71% in *Gracilaria edulis* from the southeast coast of India, as noted by Rosemary et al. (2019) [59]. For brown algae, carbohydrate levels range from 58.05% in *Gongolaria barbata* from the Black Sea, reported by Cadar E. (2017), to 9% in *Iyengaria stellata* (Phaeophyceae) from the Arabian Sea, reported by Choudhary et al. (2023) [49,53]. Additionally, carbohydrates can exist in sulfated forms, as highlighted by Sirbu et al. (2020) and Cadar et al. (2023) [30,49]. Proteins are also found in all algae, though their content varies. In green algae, protein levels range from 6.0% in *Ulva lactuca* and *Caulerpa scalpelliformis* from the Arabian Sea, reported by Choudhary et al. (2023), to 22.7% in *Ulva lactuca* (as *Ulva fasciata*), as documented by Ganesan et al. (2020) [53,55]. The protein content in red algae varies significantly, ranging from 3.0% in *Halymenia porphyriformis* from the Arabian Sea, as reported by Choudhary et al. (2023), to 29–39% in *Porphyra umbilicalis* from Atlantic waters, as noted by Morais et al. (2020) [53,54]. In brown algae, protein levels range from 3–14% in *Fucus vesiculosus* from the Atlantic, reported by Morais, to 28.02% in *Sargassum ilicifolium* from Sri Lanka, as documented by Premerathna et al. (2022) [54,58]. Lipid content is found in all algae but varies widely. In green algae, lipid levels range from 4% in *Caulerpa scalpellifera* from the Arabian Sea, as reported by Choudhary et al. (2023), to 0.76% in *Ulva flexuosa* from Indian waters, as reported by Ganesan et al. (2020) [53,55]. In red algae, lipid content varies from 0.3% in *Porphyra umbilicalis* from Atlantic waters, reported by Morais et al. (2020), to 7.07% in *Gracilaria corticata* from the southeastern coast of India, as noted by Rosemary et al. (2019) [54,59]. Brown algae show the widest lipid variation, with levels ranging from 17.06% in *Himanthalia elongata* from Sri Lanka, as recorded by Ilyas et al. (2023), to just 0.17% in *Sargassum asperifolium* from the Red Sea, reported by Fouda et al. (2019) [62,63]. Total dietary fiber is present in all algae, with green algae exhibiting the highest levels. *Caulerpa racemosa* from the coastal waters of Sri Lanka contains the most fiber, at 81.59%, as documented by Premarathna et al. (2022) [58]. Among red algae, the highest fiber content is 56.81% in *Jania pedunculata* var. *adhaerens* from Sri Lanka, also reported by Premarathna et al. (2022) [58]. For brown algae, the highest dietary fiber levels range from 43 to 59% in *Fucus vesiculosus* from the Atlantic, as studied by Morais et al. (2020) [54]. Additionally, recent studies by Ullah et al. (2024) and Xie et al. (2024) have provided valuable insights into the nutritional composition of brown algae [64,65].

### 3.4. Active Metabolites from Seaweeds

The composition of macroalgae is rich in important bioactive metabolites that are currently barely utilized for human health. The most widespread use of macroalgae has been as food. Recent studies emphasize a wide range of possibilities for the utilization of algae compositions that offer a rich range of secondary metabolites. Other researchers such as Xie et al. (2024), Choudhary et al. (2021) have also evaluated the existence and biological activities of metabolites in macroalgae [65,66].

#### 3.4.1. Polysaccharides (MAPs)

Marine algae polysaccharides (MAPs) are vital nutritional components found in all algae, serving as an energy source for the algal body. Based on molecular size and complexity, they are categorized into monosaccharides, disaccharides, oligosaccharides, and polysaccharides. Xie et al. (2024) identified mannose, glucose, fructose, galactose, fucose, xylose, and arabinose as the most common monosaccharides [65]. Dobrinčić et al. (2020) explored extraction technologies, as well as the isolation and structural characterization of MAPs from marine macroalgae [67].

**Figure 3:**
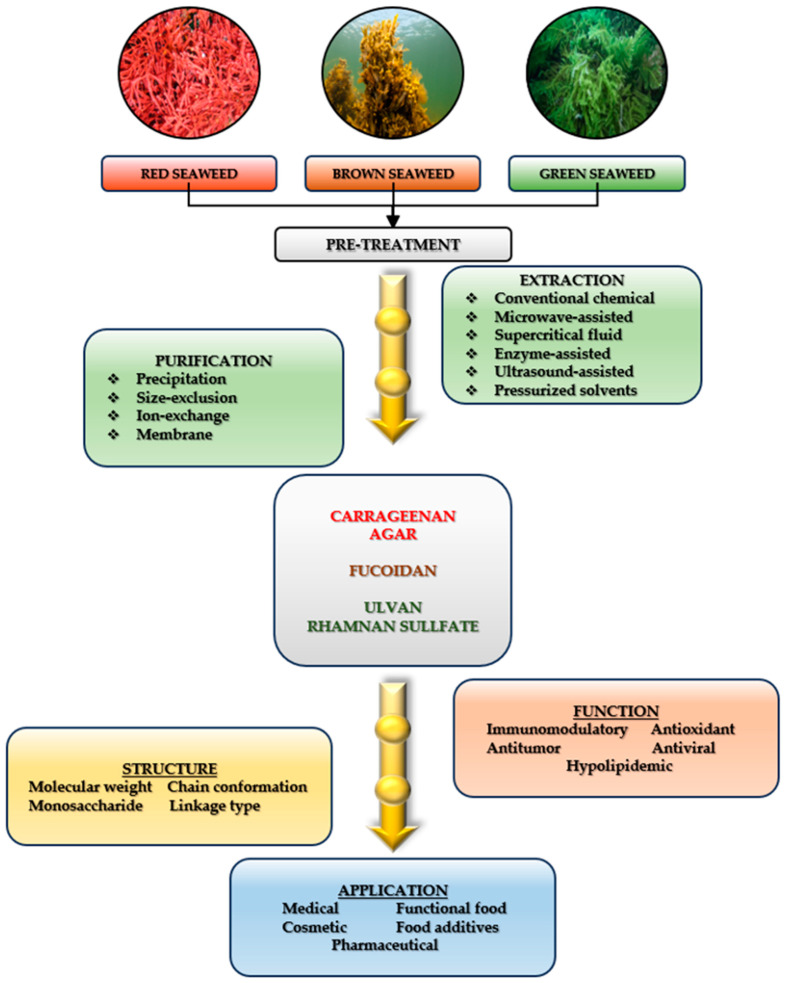
Scheme for the isolation, structural and functional analyses, and applications of polysaccharides.

Additionally, Premarathna et al. (2024) investigated how different extraction methods influence the physicochemical properties and antioxidant activity of polysaccharides in red algae [68]. The extraction of polysaccharides from seaweed and their application as hydrogels were explored by Lin et al. (2022), who demonstrated that polysaccharide composition and structure vary based on factors such as species, harvesting season, collection sites, and water quality [69]. Additionally, the extraction and purification conditions significantly impact the properties of polysaccharides and the hydrogels derived from them [69]. Ummat et al. (2021) emphasized the importance of pre-treatment steps conducted before extraction [70]. Oh et al. (2020) proposed improvements to the extraction of bioactive compounds like polysaccharides by moving away from traditional water and organic solvent-based methods, instead advocating for enzyme-assisted extraction techniques, which offer lower energy consumption and enhanced metabolite quality [71]. Yao et al. (2020) highlighted the structural diversity of polysaccharides and their varying effects depending on the algal category, while Akter et al. (2024) detailed specific polysaccharide structures with biomedical applications, including antiviral potential [72,73]. Figure 3 illustrates the polysaccharide extraction process, including pre-treatment steps.

**Figure 4:**
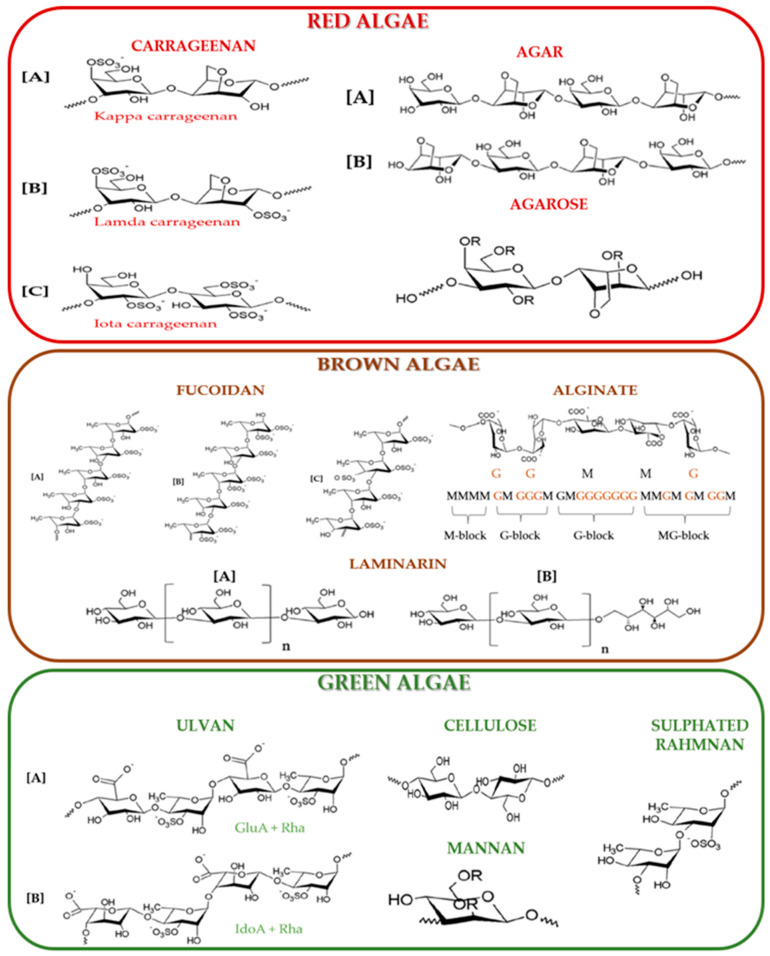
Chemical structure of polysaccharides from seaweed.

The key polysaccharide structures are depicted in Figure 4.

Carrageenans are polysaccharides found in red algae, alongside agar and agarose. Carrageenans are anionic, sulphated galactans, with their structure illustrated in Figure 4. According to Xie et al. (2024), the linear chains of carrageenans consist of repeating disaccharide units of 3, 6-anhydro-galactose and D-galactose, linked by alternating 4-α-D-galactose and 3-β-D-galactose. These chains are further modified by methyl, sulfate ester, or pyruvate substitutions, with sulfate ester groups making up 15–40% depending on the specific type of carrageenan [65]. Figure 4 shows the three main commercial forms: Kappa, Lambda, and Iota. Kappa forms rigid, strong gels in the presence of potassium ions and is primarily derived from *Kappaphycus alvarezii*. Iota, which forms soft gels in the presence of calcium ions, comes mainly from *Eucheuma denticulatum*. Lambda, on the other hand, does not form gels, as shown by Udo et al. (2023) [74].

Agars are also widely used in various industries due to their excellent hydrocolloid properties. They are extracted from species like *Gelidium* spp., *Gracilaria* spp., and *Pterocladiella capillacea* [65]. Xie et al. (2024) explain that agars are hydrocolloids composed mainly of agarose and agaropectin [65].

As shown in Figure 4**,** agars are linear polysaccharides made up of alternating residues of α-(1→3)-D-galactopyranose and β-(1→4)-linked 3,6-anhydro-L-galactopyranose, with intermittent sulfate groups at the C-6 position. Like carrageenans, agars are sulphated galactans. Zhang et al. (2019) demonstrated that the anionic charges in agar polymers vary depending on the degree of sulfation [75]. Dragan et al. (2022) noted that further research is needed to understand the biosynthesis of c arrageenans [76].

Fucoidans are a key component of brown algae, along with other major MAPs such as alginates and laminarin. They belong to the group of sulfated polysaccharides, primarily composed of sulfated α-L-fucose residues, but also include glucose, xylose, mannose, galactose, uronic acids, and acetyl groups. Shao et al. (2022) demonstrated that fucoidans are a primary constituent of the cell walls in brown seaweeds, distinguishing them from terrestrial plants [77].

According to Xie et al. (2024), the fucoidan content in brown algae typically ranges from 10% to 20%, depending on factors like the species, season, reproductive cycles, environmental conditions, and tissue position. The highest concentration reported to date is 46.6% in *Laminaria digitata* [65]. Fucoidan structures mainly consist of two types of backbones: one made of α-(1→3)-L-fucose residues, and another alternating between (1→3)-linked and (1→4)-linked α-L-fucose residues [65]. Wang et al. (2020) explored the structure of fucoidans from *Sargassum siliquosum* [78], and Dragan et al. (2023) analyzed fucoidans from *Gongolaria barbata*, focusing on their potential health benefits for humans [79].

Laminarins are low molecular weight polysaccharides found specifically in brown algae. Their polymeric structure consists of a chain of (1→3)-linked β-D-glucopyranose units, with varying degrees of β-(1→6)-linkages between chains. Rajauria et al. (2021) studied laminarin rings in species such as *Saccharina* sp., *Laminaria* sp., and *Fucus* sp., identifying two primary types of polymer chains: G-chain and M-chain. These chains have a lower molecular weight compared to other seaweed polysaccharides and vary based on the degree of polymerization [80]. Li et al. (2021) explained that the distinction between the two chains lies in the presence of D-mannitol at the reducing end of the M-chain, whereas the G-chain lacks D-mannitol at this position [81].

Alginates are the predominant polysaccharides found in the intercellular matrix and cell walls of brown algae. Xie et al. (2024) reported that alginate content is highest in young algae during July, varying between 17% and 47% [65]. Shao et al. (2022) described alginates as linear anionic polymers composed of α-L-guluronic acid (G) and β-D-mannuronic acid (M) units, linked by (1→4) glycosidic bonds [77]. Tanna et al. (2019) also confirmed that these isomeric residues are connected by (1→4) glycosidic bonds [82]. Ramos et al. (2018) noted that the M/G ratio can be influenced by factors such as species, growth conditions, harvesting time, and extraction methods [83]. Pengyan et al. (2021) showed that the gel stiffness follows this order: gel formed from homo-polymeric G blocks > gel formed from homo-polymeric M blocks > gel formed from hetero-polymeric MG blocks [84]. Abka-khajouei et al. (2022) evaluated the structural characteristics of alginates from seaweed and highlighted their diverse applications [85].

Ulvans, the most abundant polysaccharides found in green algae, include ulvan, cellulose, mannan, and sulfated rhamnan. The structure of ulvan has been studied by Glasson et al. (2022), Li Q et al. (2020), and Sari-Chmayssem et al. (2019) [86,87,88]. Ulvan is a polyanionic heteropolysaccharide composed of uronic acids (iduronic acid and glucuronic acid), rhamnose 3-sulfate, and xylose. Tanna et al. (2019) demonstrated that ulvan has a complex, heterogeneous composition, featuring repeating disaccharide units, such as xylose, sulfated rhamnose, and uronic acids (iduronic or glucuronic acid) [82]. Xie et al. (2024) further clarified that sulfated rhamnose residues typically occupy the C-3 position or both C-1 and C-3 positions, while sulfated xylose residues may replace uronic acids [65]. Additional studies on the structures of green algae polysaccharides were conducted by Ciancia et al. (2020) and Gomaa et al. (2022) [89,90]. The polysaccharide content was presented for representative algae in Table 1 where the nutritional composition of macroalgae was demonstrated.

Researchers have also focused on optimizing the extraction of these valuable biocompounds. Carrageenans have been extracted from various red algae by Firdayanti et al. (2023), Martín-del-Campo et al. (2021), and Heriyanto et al. (2018) [91,92,93]. Firdayanti et al. (2023) reported the highest extraction yield using bead mill extraction, while the lowest yield was obtained by Heriyanto et al. (2018) using conventional extraction methods [91,93]. Agar extraction from red algae species has been explored by Lebbar et al. (2018), Martínez-Sanz et al. (2019), and Xiao et al. (2019) through various methods [94,95,96]. The highest yield was achieved using alkaline extraction by Xiao et al. (2019) from *Gracilariopsis lemaneiformis* (formerly *Gracilariopsis lemaneiformis*) (Rhodophyta) [96]. Table 2 presents different polysaccharide types alongside the optimal yields achieved through various extraction methods. Fucoidan extraction has been studied using alternative methods by Alboofetileh et al. (2018), Hmelkov et al. (2018), Alboofetileh et al. (2019), Liu et al. (2020), and Hanjabam et al. (2019) from different brown algae species [97,98,99,100,101]. The highest extraction efficiency was reported by Hanjabam et al. (2019), who employed the UAE method for isolating fucoidans from *Sargassum wightii*, a brown algae species [101]. Alginates have been extracted by Montes et al. (2021), Rashedy et al. (2021), and Trica et al. (2019) [102,103,104]. Finally, ulvan extraction from different *Ulva* species was conducted by Malvis Romero et al. (2023), Kidgell et al. (2019), Yuan et al. (2018), and Tabarsa et al. (2018) [105,106,107,108].

The highest extraction yield for ulvan was reported by Kidgell et al. (2019) by conventional extraction, followed by Yuan et al. (2018) by microwave-assisted hydrothermal extraction, who extracted ulvan from various species of *Ulva* sp. and by different methods [106,107]. Table 2 presents different polysaccharide types alongside the optimal yields achieved through various extraction methods.

#### 3.4.2. Terpenoids Content

**Figure 5:**
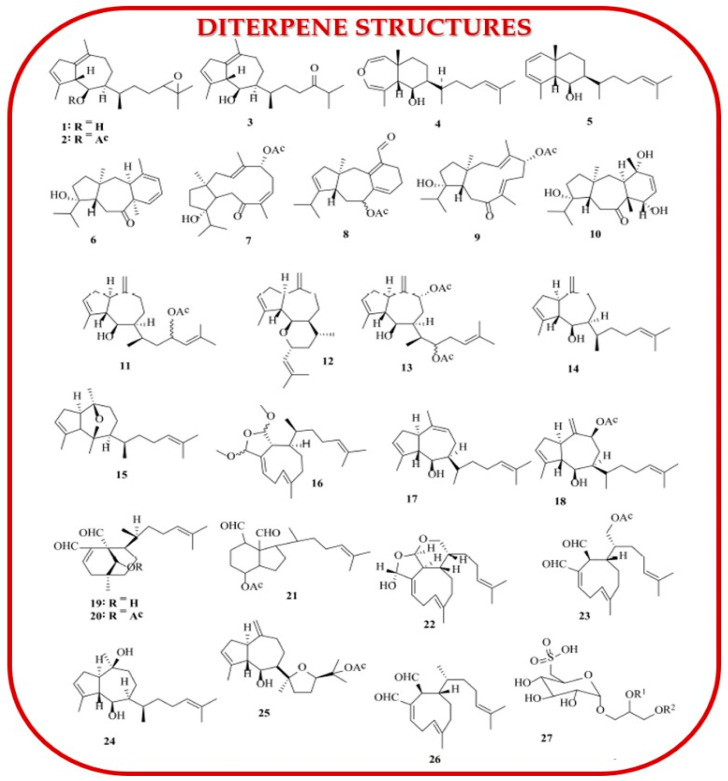
Structure of diterpenes from species of the genus *Dictyota*.

Terpenic compounds, along with other secondary metabolites, are significant in marine macroalgae. These compounds include monoterpenes, sesquiterpenes, diterpenes, and triterpenes. Structurally, terpenes are a large and diverse group of compounds with the general formula (C_5_H_8_)_n_, composed of isoprene units (2-methylbuta-1,3-diene). They are found not only in marine algae but also in the volatile oils of terrestrial plants, as noted by Cikoš et al. (2019) [109]. Polzin et al. (2018) focused on monoterpenes from red algae of the genus *Ochtodes*, particularly *Ochtodes secundiramea* [110]. Figure 5 displays the chemical structures of the diterpenes identified from several *Dictyota* species.

Diterpenes, another significant category, are a large and structurally diverse class of compounds found widely in marine macroalgae. Chen et al. (2018) highlighted the strong cytotoxic and antiviral properties of diterpenes [111]. They identified diterpenes 1–5 from *Dictyota acutiloba*, diterpenes 6–10 from *Dictyota bartayresiana*, diterpenes 11–16 from *Dictyota binghamiae*, diterpenes 17–21 from *Dictyota caribaea*, and diterpenes 22–26, along with sulfonoglycolipid 27, from *Dictyota ciliolata* (Phaeophyceae). Monoterpenes were extracted through conventional chemical methods by Cikoš et al. (2019), who also examined the influence of solvents on extraction and the interference of other compounds with monoterpenes during GC-MS analysis [109]. Cikoš et al. (2019) also extracted halogenated monoterpenes from *Plocamium cartilagineum* using supercritical fluid extraction [109]. Their work focused on red algae from the genera *Plocamium* and *Portieria*, which are rich in cyclic and acyclic halogenated monoterpenes, demonstrating their role as antitumor agents [109]. Polzin et al. (2018) also investigated the extraction of halogenated monoterpenes from *Ochtodes* species [110]. Furthermore, Chen et al. (2018) reported that, by the end of 2017, a total of 233 diterpenes had been isolated from *Dictyota* species, particularly from the brown alga *Dictyota dichotoma* [111].

Rajamani et al. (2018) conducted studies on the extraction and characterization of triterpenes isolated from the brown alga *Padina boergesenii* [112]. Using microwave-assisted extraction (MAE), Nie et al. (2021) identified three novel terpenoids from the brown seaweed *Sargassum fusiforme* [113]. Rushdi et al. (2022) reported a large-scale study over a longer period (1976–2022) on bioactive compounds isolated from brown seaweeds of the genus *Dictyota* and indicated that numerous compounds such as diterpenes and sesquiterpene exhibited various biological activities [114].

#### 3.4.3. Seaweeds Lipids, Fatty Acids (AFs), and Sterols

Santos et al. (2019) reported the lipid profiles of three seaweed species from Brazilian marine waters using a modified Folch method, revealing that glycolipids were the most abundant lipid class in all species, making up 60–70% of the total lipids [115]. This was followed by phospholipids (10–25%) and neutral lipids (10–15%) [115]. Table 3 provides a detailed breakdown of fatty acid content by fatty acid structure categories for several representative macroalgae species from the three major classes: green, red, and brown. Peñalver et al. (2020) also highlighted the main lipid classes in macroalgae, including neutral lipids (fatty acids, triglycerides, and sterols), glycolipids, and phospholipids [116].

Foseid et al. (2020) reported lipid extraction studies using a modified Folch method, which involved extraction followed by GC-MS analysis of lipids from the red alga *Parmaria palmata* and two brown algae species, *Alaria esculenta* and *Saccharina latissima* [117]. Rocha et al. (2021) investigated the composition and concentration of lipids in macroalgae, finding significant variation in lipid content across species, which was influenced by season, temperature, geographical area, and environmental conditions [118]. El-Sheekh et al. (2021) demonstrated lipid extraction using the modified Folch method on algae harvested from the Abu Qiu Bay area in Egypt. They found that the highest lipid content occurred in the spring season for *Ulva fascinata* (14.66%) and *Ulva compressa* (9.94%) [119]. Jaworowska et al. (2023) highlighted that macroalgae are a rich source of biologically active lipids, particularly unsaturated fatty acids [120]. Kord et al. (2019) examined the fatty acid content of *Gongolaria sauvageauana* (formerly *Cystoseira sauvageauana*) (Phaeophyceae) and *Osmundea pinnatifida* (formerly *Laurencia pinnatifida*) (Rhodophyta), algae collected from the Algerian coast [121]. Their methods for determining SFA, MUFA, PUFA, and the ω-6/ω-3 ratio were later expanded upon by Rodriguez-Gonzalez et al. (2022), who conducted a more comprehensive study [121,122]. Rodriguez-Gonzalez et al. (2022) also explored several alternative extraction techniques for fatty acid analysis, including ultrasound-assisted extraction (UAE), enzyme-assisted extraction (EAE), pulsed electric fields-assisted extraction (PEF), pressurized liquid extraction (PLF), microwave-assisted extraction (MAE), and supercritical CO_2_ extraction (Sc-CO_2_) [122]. The fatty acid content of algae has been widely studied. Susanto et al. (2019) and Pangestuti et al. (2021) also presented findings on the fatty acid content of green marine macroalgae [123,124]. Lopes et al. (2020) explored the role of lipids in aquaculture red macroalgae for various biotechnological applications [125], while Lopes et al. (2019) reported the lipid content and properties of the red alga *Palmaria palmata* [126]. Al-Adilah et al. (2021) provided additional nutritional information, including fatty acid content, for various brown macroalgae species [127]. According to Rocha et al. (2021) and Jaworowska et al. (2023), the percentage of saturated fatty acids (SFAs) ranged from 7.53% to 95.21%, monounsaturated fatty acids (MUFAs) ranged from 2.30% to 47.10%, and polyunsaturated fatty acids (PUFAs) ranged from 2.60% to 73.70% [118,120]. Among fatty acids, palmitic acid is the most prevalent, followed by oleic acid, based on their percentages in the total fatty acid profile, as reported by Jaworowska et al. (2023) [120]. Both Jaworowska et al. (2023) and Harwood et al. (2019) demonstrated that seaweeds are an important source of essential PUFAs, such as acids ALA, C18:3, and n-3 and acids LA, C18:2, and n-6, which mammals cannot synthesize [120,128]. According to Jaworowska et al. (2023), green algae have the lowest ω-6/ω-3 ratio, followed by brown algae, with red algae exhibiting the highest ratio [120]. It is important to note that significant amounts of docosahexaenoic acid (DHA) and eicosapentaenoic acid (EPA) are found in macroalgae, as humans have limited endogenous synthesis of these acids. Table 3 also highlights the omega-6/omega-3 ratio, which impacts human health by reducing the risk of cardiovascular, neurological, and inflammatory diseases. Of the two, EPA is the dominant (n-3) LC-PUFA and its concentration exceeds that of DHA, as shown in Table 3.

**Table 3:** Fatty acid content, reported for some representative seaweeds for each of the three classes: green, red, and brown macroalgae.

Green Algae					Red Algae				Brown Algae				
Fatty Acids	*Cladophora albida*	*Ulva intestinalis*	*Caulerpa lentillifera*	*Ulva lactuca*	*Ceramium deslongchampsii* (as *Ceramium strictum)*	*Gracilaria gracilaris*	*Porphyra dioica*	*Palmaria palmata*	*Stephanocystis hakodatensis* (as *Cystoseira hakodatensis*)	*Dictyota dichotoma*	*Undaria pinnatifida*	*Sargassum horneri*	*Padina boergesenii*
C16:0	33.04 ± 0.52	31.05 ± 11.10	33.69 ± 0.64	33.39 ± 12.87	24.00 ± 0.60	27.1 ± 1.2	25.71 ± 3.40	24.32 ± 1.11	18.49 ± 0.30	24.75 ± 0.32	11.51 ± 0.01	25.24 ± 1.91	49.20 ± 0.30
C18:0	1.28 ± 0.19	n.d.a	13.57 ± 0.91	2.21 ± 1.32	3.30 ± 0.20	4.6 ± 0.8	3.53 ± 1.93	12.45 ± 6.74	n.d.a	2.85 ± 0.08	0.64 ± 0.02	28.90 ± 2.33	2.30 ± 0.10
∑SFA	50.03 ± 0.56	42.80 ± 25.17	29.80 ± 1.65	54.95 ± 26.77	34.80	34.9 ± 0.9	34.32 ± 4.49	36.77 ± 6.95	n.d.a	35.98 ± 0.47	12.15	26.98 ± 0.00	58.00 ± 0.40
C16:1	13.90 ± 0.09	4.85 ± 4.31	n.d.a	3.31 ± 3.64	7.30 ± 0.30	2.8 ± 0.8	10.78 ± 11.90	2.03 ± 0.43	0.63 ± 0.08	15.49 ± 0.09	1.66 ± 0.06	n.d.a	3.20 ± 0.30
C18:1	12.51 ± 0.02	12.35 ± 4.03	n.d.a	8.88 ± 4.49	18.30 ± 0.20	9.7 ± 0.4	2.85 ± 0.63	2.82 ± 0.54	11.08 ± 0.24	8.49 ± 0.13	6.21 ± 0.11	n.d.a	16.80 ± 0.80
∑MUFA	27.73 ± 0.11	23.60 ± 1.69	9.08 ± 2.75	15.45 ± 7.71	30.30	12.5 ± 0.7	13.63 ± 9.94	4.85 ± 0.56	n.d.a	24.28 ± 0.13	10.35	14.24 ± 0.00	20.50 ± 0.80
C18:2(LA)	15.54 ± 0.22	7.10 ± 1.83	n.d.a	5.54 ± 3.57	2.00 ± 0.10	2 ± 0.4	1.67 ± 0.04	0.45 ± 0.19	6.95 ± 0.15	5.55 ± 0.02	3.87 ± 0.08	n.d.a	3.30 ± 0.10
C18:3 (ALA)	n.d.a	7.85 ± 9.26	n.d.a	5.64 ± 6.03	15.10 ± 0.30	2.7 ± 0.2	1.95 ± 0.07	n.d.a	6.87 ± 0.18	2.63 ± 0.20	19.84 ± 0.42	n.d.a	2.20 ± 0.1
∑PUFA	16.24 ± 0.24	25.95 ± 15.76	13.06 ± 0.32	17.62 ± 19.64	24.90	5.26 ± 1.4	13.78 ± 12.42	0.45 ± 0.19	n.d.a	19.74 ± 0.67	23.71	29.00 ± 0.00	6.40 ± 0.50
C20:4(ARA)	1.37 ± 0.07	n.d.a.	2.84 ± 0.53	2.50 ± 4.03	3.90 ± 0.10	35.4 ± 1.5	3.16 ± 0.19	0.92 ± 0.17	16.59 ± 0.11	11.46 ± 0.59	0.00	23.04 ± 0.52	2.0 ± 0.1
C20:5(EPA)	2.02 ± 0.05	0.55 ± 0.35	n.d.a	1.69 ± 1.12	n.d.a	5.5 ± 0.2	33.42 ± 18.27	51.68 ± 6.47	12.96 ± 0.18	6.57 ± 0.22	13.15 ± 0.02	n.d.a	0.3 ± 0.02
C22:6 (DHA)	0.86 ± 0.03	n.d.a	n.d.a	0.65 ± 0.63	n.d.a	n.d.a	n.d.a	n.d.a	n.d.a	n.d.a	8.55 ± 0.37	n.d.a	n.d.a
∑HUFA	4.25	0.55	2.84	4.84	3.90	40.9	36.58	52.6 ± 6.40	29.55	18.03	21.70	23.04	2.3
∑FA	98.25	92.9	54.78	92.86	93.90	93.56	98.31	3578	n.d.a	98.03	68.11	93.26	87.2
ω-6/ω-3	6.73	0.35 ± 0.21	0.79 ± 0.05	0.77 ± 0.48	n.d.a	2.47	1.22 ± 1.49	-	1.32	3.52	67.86	0.99 ± 0.30	1.4 ± 0.03
References	[120]	[120]	[123]	[120,124]	[120]	[125]	[125]	[126]	[120]	[120]	[118]	[123]	[127]

Results are expressed in mean % ± standard deviation. (LA)—linoleic acid; (ALA)—alpha linoleic acid; (ARA)—arachidonic acid; (DHA)—docosahexaenoic acid; (EPA)—eicosapentaenoic acid. (∑FA) content is the sum of fatty acid. This includes (∑SFA)—saturated fatty acids; (∑MUFA)—monounsaturated fatty acids; (∑PUFA)—polyunsaturated fatty acids. (∑HUFA)—highly unsaturated fatty acids. ω-6/ω-3 is the ratio of ω-6/ω-3.

The percentage of EPA in total fatty acids decreases in the order of Rhodophyta, Heterokontophyta, and Chlorophyta, with the lowest EPA content in Chlorophyta. Similarly, DHA content follows the same decreasing order: Rhodophyta, Heterokontophyta, and Chlorophyta.

#### 3.4.4. Proteins and Amino Acids

Seaweeds are a significant source of proteins, as well as key ingredients in food, nutraceuticals, and functional foods for both humans and animals. Pliego-Cortésa et al. (2020) reported that seaweeds contain valuable protein content, reaching up to 50% of their dry weight (d.w.), including peptides, enzymes, glycoproteins, lectins, amino acids with mycosporine structures, and phycobiliproteins found in red algae [129]. Similarly, Dhaouafi et al. (2024) highlighted that marine macroalgae serve as an important reservoir of biologically active compounds such as proteins, peptides, and amino acids, along with enzymes, carotenes, fatty acids, flavonoids, vitamins, minerals, and polysaccharides [130]. The protein content varies among different algae species, with studies by Pliego-Cortésa et al. (2020) and Fleuence et al. (2018) indicating that red algae have the highest protein content (20–47%), followed by green algae (9–26%) and brown algae (3–15%) [129,131]. Rawiwan et al. (2022) further emphasized that the high protein content (up to 47%) and essential amino acids (EAAs) in red seaweeds make them a valuable protein source [132]. Beyond their nutritional value, seaweed proteins also exhibit bioactive properties. Feng et al. (2021) investigated peptide proteins from the brown alga *Undaria pinnatifida*, which demonstrated antihypertensive activity [133]. However, Pliego-Cortésa et al. (2020) warned that seaweeds contain sources of non-protein nitrogen, such as nitrite, pigments, and nucleic acids, which may lead to an overestimation of the protein content calculated with the conversion factor of 6.25 [129]. To address this, their analysis of 44 studies covering 103 algae species led to the proposal of new seaweed nitrogen–protein conversion factors (SNPs): 5.38 for brown algae, 4.59 for red algae, and 5.13 for green algae [129]. Various protein types have been identified in seaweeds.

Peptides, which are protein fragments consisting of 3 to 40 amino acids, are one such category. Marine algae also contain enzymes, including alkaline phosphatase, a zinc-containing metalloproteinase that catalyzes the hydrolysis of phosphate monoesters, as demonstrated by Pliego-Cortésa et al. (2020) in extracts from *Ulva australis* (formerly *Ulva pertusa*) (Chlorophyta) [129]. Ünlü et al. (2019) identified alternative oxidase proteins (AOXs) in *Caulerpa cylindracea*, which contribute to the alga’s ability to invade new environments [134].

Glycoproteins (GPs). Another key group is glycoproteins (GPs), which consist of proteins covalently linked to oligosaccharide chains. Echave et al. (2022) classified these chains into two major types: those linked by O-glycosyl bonds and those linked by N-glycosyl bonds [135]. Lectins, a class of proteins that bind specifically to mono- and oligosaccharides, are particularly abundant in red algae. Echave et al. (2022) highlighted that marine lectins are predominantly mannose-specific and exhibit strong binding affinities [135]. Barre et al. (2019) noted that lectins play crucial roles in reproductive cell fusion, pathogen defense, and possess significant anti-cancer properties [136].

**Figure 6:**
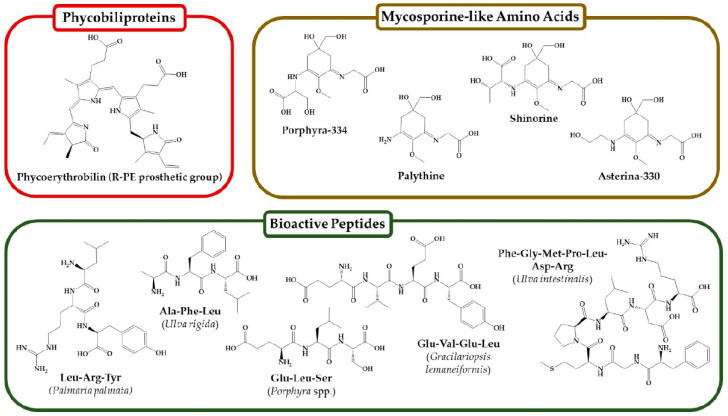
Structures for phycobiliproteins, mycrosporine-like amino acids, and bioactive peptides.

Arabinogalactan proteins (AGPs). Additionally, some proteins are associated with the algal cell wall, such as arabinogalactan proteins (AGPs), which are glycoproteins rich in hydroxyproline. While AGPs are well-studied in terrestrial plants, their role in marine algae remains less understood. According to Pliego-Cortésa et al. (2020), the carbohydrate portion of AGPs (90–95%) consists of arabinose, rhamnose, and glucuronic acid residues, while the protein backbone is composed of hydroxyproline/proline, alanine, and serine/threonine sequences [129]. Figure 6 illustrates the structures of key protein classes found in seaweeds, including phycobiliproteins, mycosporine-like amino acids, and significant bioactive peptides.

Phycobiliproteins (PBPs) are the main pigments that play a role in light capture, and are the only water-soluble pigments. Pliego-Cortésa et al. (2020) show that PBPs are made up of four classes: phycoerythrin (PE), phycocyanin (PC), phycoerythrocyanin (PEC), and allophycocyanin (APC); Phycoerythrin (PE) is the main pigment and is divided into R-for hodophyta (R-PE) and B-for Bingiales (B-PE) [129].

Mycosporine-like Amino Acids (MAAs). These compounds are small in size (<400 Da), are secondary metabolites with strong absorption in UVR, and are stable under variable light conditions, temperature, and pH. Pliego-Cortésa et al. (2020) show that they have main functions in UVR protection and antioxidant activity, and facilitate the adaptation of algae to stressful environments [129].

Extraction of peptides from seaweed. The extraction of peptides from seaweed requires several pre-treatment steps to enhance efficiency. Echave et al. (2021) outlined common pre-treatment methods, including osmotic shock, mechanical grinding, alkaline treatment, freeze–thaw cycles, and ultrasonic sonication [135]. Protein extraction techniques include solid–liquid extraction (SLE), microwave-assisted extraction (MAE), pulsed electric field (PEF), ultrasound-assisted extraction (UAE), enzyme-assisted extraction (EAE), and high hydrostatic pressure extraction (HHPE). According to Echave et al. (2021), pre-treatment simplifies the extraction process and improves yield, while protein hydrolysis facilitates the production of bioactive peptide-rich protein hydrolysates (BAPs) [135]. Vásquez et al. (2019) explored enzyme-assisted protein extraction from *Macrocystis pyrifera* (Phaeophyceae) and *Chondracanthus chamissoi* (Rhodophyta) [137]. O’Connor et al. (2020) compared three physical extraction methods applied to four algae species: two red (*Palmaria palmata* and *Chondrus crispus*) and two brown (*Fucus vesiculosus* and *Alaria esculenta*). Their study highlighted the challenge of breaking the algal cell wall to release proteins efficiently [138]. They also found that combining heat and pressure (as in the autoclave method) resulted in the highest extraction yields for *Palmaria palmata*, while physical pre-treatment increased the essential amino acid (EAA) content of extracts compared to raw biomass [138].

Amino acid composition varies among seaweed species. Vieira et al. (2018) studied amino acids in the green alga *Ulva rigida* and several brown algae [139]. Further research on *Ulva rigida* was conducted by Sonchaeng et al. (2023) and Machado et al. (2020) [140,141]. For red algae, amino acid compositions were analyzed by O’Connor et al. (2020) for *Palmaria palmata* and *Chondrus crispus* and by Machado et al. (2020) for *Porphyra dioica* [138,141]. Ferreira et al. (2021) and Trigueros et al. (2021) investigated the amino acid profiles of *Gracilaria gracilis* and *Gelidium corneum* [142,143]. Regarding brown algae, Vieira et al. (2018) reported on *Fucus spiralis*, *Ascophyllum nodosum*, and *Undaria pinnatifida*, while Zheng et al. (2020) analyzed *Sargassum mcclurei* [139,144]. O’Connor et al. (2020) reported that *Chondrus crispus* has the highest protein content (35.2% d.w.), while Sonchaeng et al. (2023) found that *Ulva lactuca* has the lowest (5.67% d.w.) [138,140]. Ferreira et al. (2021) recorded the highest EAAs percentage in *Gracilaria gracilis* (45.6% d.w.) and Zheng et al. (2020) reported the lowest in *Sargassum mcclurei* (27.8% d.w. of protein content) [142,144]. Reynolds et al. (2022) emphasized the nutritional value of seaweed proteins for human diets [145]. Hydroxylysine (*Hyl*) has not been detected in marine algae, except in *Palmaria palmata*, where O’Connor et al. (2020) reported a concentration of 2.7% of total amino acids [138]. Hydroxyproline (*Hyp*) is mainly found in brown algae, as noted by Vieira et al. (2018) for *Fucus spiralis*, *Ascophyllum nodosum*, and *Undaria pinnatifida* [139]. Table 4 summarizes amino acid compositions across various algae.

#### 3.4.5. Pigments Content from Seaweeds

Marine algae produce pigments as secondary metabolites, which have significant bioactive properties and diverse applications in the food, pharmaceutical, and cosmetic industries. Extensive research has explored these pigments. Manzoor et al. (2024) demonstrated that marine algal pigments exhibit therapeutic benefits, including anti-cancer, antioxidant, anti-obesity, neuroprotective, anti-inflammatory, and anti-angiogenic activities [146]. Marine algal pigments are classified into three major groups: chlorophylls, carotenoids, and phycobiliproteins.

Chlorophylls are fat-soluble pigments essential for light harvesting, electron transport, and energy transmission in photosynthesis. Their chemical structure consists of a substituted porphyrin ring (acting as a chelating ligand) and a phytol carbon chain. The primary types of chlorophylls in seaweed are chlorophyll a, b, and c. Gomes et al. (2022) identified chlorophyll a (C_55_H_72_MgN_4_O_5_) as the most abundant pigment in seaweed, exhibiting antimicrobial potential and a blue-green color with a maximum absorption range of 660–665 nm [147]. Aryee et al. (2018) described chlorophyll b (C_55_H_70_MgN_4_O_6_) as the second most important, exclusive to green algae, and appearing green-yellow with an absorption range of 642–652 nm, relevant in food processing [148]. Chlorophyll c, found in brown algae, is a blue-green pigment with an absorption range of 447–452 nm and antimicrobial properties as shown by Gomes et al. (2022) [147]. It has three variants: chlorophyll c 1 (C_35_H_30_MgN_4_O_5_, absorption peak at 447 nm), chlorophyll c 2 (C_35_H_28_MgN_4_O_5_, absorption peak at 450 nm), and chlorophyll c 3, which is absent in marine algae [147].

Carotenoids, ranging in color from yellow to orange-red, have tetrapenoid structures that aid photosynthesis and are most abundant in brown algae as shown by Pérez-Gálvez et al., 2020 [149]. Manzoor et al. (2024) classified carotenoids into two groups based on molecular structure: xanthophylls (oxygen-containing) and hydrocarbons (carotene-based) [146]. According to Gomes et al. (2022), xanthophylls include fucoxanthin, astaxanthin, lutein, lorxanthin, violaxanthin, neoxanthin, and zeaxanthin, while carotenes lack oxygen atoms [147].

Phycobiliproteins (PBPs) are water-soluble, non-toxic proteins with high physiological stability. They are divided into three groups: phycoerythrins, allophycocyanins, and phycocyanins. Manzoor et al. (2024) found that PBPs constitute up to 60% of the soluble protein in cyanobacterial cells [146]. Cotas et al. (2020) reported that PBP absorption ranges from 450 to 570 nm, with B-phycoerythrin peaking at 499, 546, and 565 nm, C-phycoerythrin at 565 nm, and R-phycoerythrin at 498, 540, and 565 nm [150]. Ghosh et al. (2022) highlighted the importance of phycoerythrins as red-colored functional food ingredients [151]. Manivasagan et al. (2018) described phycocyanin as containing chromophore phycobilins, capturing blue light, and having an absorption range of 610–620 nm, with various therapeutic applications [152].

Regarding pigment extraction, Osório et al. (2020) investigated the separation of chlorophylls, fucoxanthin, and phycobiliproteins using conventional solvent-based extraction from commercial seaweeds, including brown algae (*Himanthalia elongata*, *Undaria pinnatifida*, *Laminaria ochroleuca*) and red algae (*Porphyra* spp.) [153]. The study found that extraction efficiency varied by solvent, with acetone yielding the highest chlorophyll extraction from brown algae, while methanol was most effective for red algae. Notably, *Porphyra* spp. contained significantly higher pigment levels than brown algae [153]. Manzoor et al. (2024) explored various green extraction methods for obtaining pigments from algae, including microwave-assisted extraction (MAE), pressurized liquid extraction (PLE), and supporting their supercritical fluid extraction (SFE) [146]. These techniques offer ecological benefits such as lower operating temperatures, shorter extraction times, reduced use of chemical solvents, and automation that enhances compound recovery.

**Figure 7:**
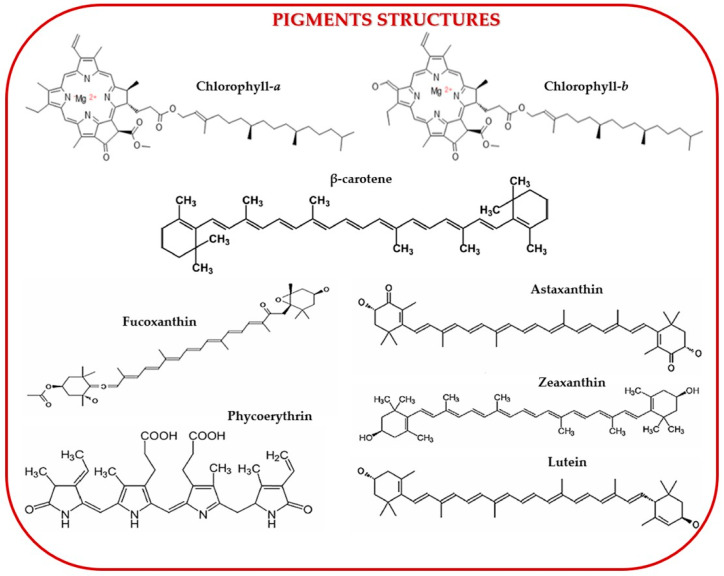
The structure of clorophyll-a, clorophyll-b, β-carotene, fucoxanthin, astaxantin, zeaxanthin, phycoerythtin, and lutein.

Figure 7 illustrates the structures of commonly found seaweed pigments.

Fabrowska et al. (2018) and Martins et al. (2021) successfully extracted chlorophyll from green algae using the MAE method [154,155]. Similarly, Nie et al. (2021) reported the extraction of carotenoid pigments through ultrasound-assisted extraction (UAE) [156]. Carreira-Casais et al. (2021) conducted a comprehensive study on extracting bioactive compounds—including fucoxanthin, chlorophylls, β-carotene, polysaccharides, and phenolic compounds—from food-grade marine algae using UAE [157]. Brain-Isasi et al. (2022) also used UAE to obtain total phycobiliproteins from *Gracilaria chilensis* [158]. Additionally, Ktari et al. (2021) employed SFE to extract fucoxanthin from *Dictyopteris polypodioides* [159]. Another alternative approach, reported by Martinez et al. (2019), involved pulsed electric field (PEF) extraction of carotenoids from *Haematococcus pluvialis* [160]. Despite these advancements, alternative extraction methods remain under development and require further optimization. As Manzoor et al. (2024) highlighted, selecting an appropriate solvent is crucial for maximizing pigment extraction efficiency [146]. Several studies have highlighted the presence of pigments in green algae, emphasizing their role as secondary metabolites. Cadar et al. (2023) identified chlorophyll-α, chlorophyll-b, and total carotenoids in green algae from the Romanian Black Sea coast, specifically *Ulva lactuca*, *Ulva intestinalis*, and *Cladophora vagabunda*, supporting their antioxidant activity [49]. The total chlorophyll and total carotenoid content in algae have been widely studied by researchers such as Sirbu et al. (2020), Choudhary et al. (2023), and Ganesan et al. (2020) [30,53,55].

The identification and quantification of pigments were documented by the respective authors, with results summarized in Table 5, categorizing pigment content across green, red, and brown algae.

Various species have been analyzed across different marine habitats and time periods to assess pigment composition. For instance, pigment values, including total chlorophyll, chlorophyll-a, chlorophyll-b, total carotenoids, and β-carotene, were reported for *Ulva lactuca* (formerly *Ulva fascinata*) from Indian waters and the coastal areas of the Philippines by Ganesan et al. (2020) and Magdugo et al. (2020) [55,161]. Similarly, pigment data for *Ulva intestinalis* from the Black Sea (Romanian coast) and Indian waters were documented by Sirbu et al. (2020) and Cadar et al. (2023) [30,49]. Additionally, *Ulva flexuosa* was studied by Ganesan et al. (2020) for the same pigment types [55]. Choudhary et al. (2023) reported pigment data, including total chlorophyll, chlorophyll-a, chlorophyll-b, total carotenoids, and β-carotene, for *Acrosiphonia orientalis* and *Caulerpa scalpelliformis* from the Saurashtra coast [53]. Magdugo et al. (2020) and Palaniyappan et al. (2023) examined *Caulerpa racemosa* from the coastal areas of the Philippines and Indian waters, respectively, documenting its pigment composition [161,162]. Kurniawan et al. (2023) identified additional pigments in *Caulerpa racemosa* from the Indonesian coast, including a high quantity of β-carotene (20.5 mg/g) alongside smaller amounts of fucoxanthin, astaxanthin, zeaxanthin, and lutein [163]. Other studies also reported pigment data from different algae species. Othman et al. (2018) analyzed *Caulerpa lentillifera* from Malaysian waters, and reported values for total chlorophyll, chlorophyll-a, chlorophyll-b, total carotenoids, β-carotene, zeaxanthin, and lutein [164]. Similarly, Babadi et al. (2020) reported total chlorophyll, chlorophyll-a, chlorophyll-b, total carotenoids, and lutein in *Chlorococcum infusionum* (formerly *Chlorococcum humicola*) (green microalga) from the Thai coast [165]. Among green algae, *Caulerpa racemosa* from the Philippines showed the highest total chlorophyll content (123.58 mg/g) according to Magdugo et al. (2020) [161]. The highest total carotenoid content (63.47 mg/g) was found in *Caulerpa lentillifera* (Chlorophyta), followed by *Kappaphycus striatum* (Rhodophyta) (57.02 mg/g) from Malaysian waters, as reported by Othman et al. (2018) [164].

Regarding red algae, Bhat et al. (2021) extracted carotenoids from *Gracilaria corticata*, while Balasubramaniam et al. (2020) identified β-carotene, fucoxanthin, astaxanthin, zeaxanthin, and lutein in eucheuma denticulatum from Malaysian waters [166,167]. The lowest total carotenoid content was reported in *Gracilaria edulis* (0.13 ± 0.02 mg/g) by Ganesan et al. (2020), while the highest was found in *Kappaphycus striatus* (57.02 mg/g) as shown by Othman et al. (2018) [55,164]. In brown algae, fucoxanthin is the dominant pigment alongside chlorophylls and β-carotene. The highest fucoxanthin content was found in *Saccharina latissima* (formerly *Laminaria saccharina*) (Phaeophyceae) (9.54 mg/g), followed by *Undaria pinnatifida* (6.15 mg/g) from the Galician coast of Spain, as reported by Lourenço-Lopes et al. (2022) [168]. Othman et al. (2018) studied *Padina pavonica* and found significant levels of total carotenoids (100.89 mg/g) along with zeaxanthin and lutein [164]. Osório et al. (2020) analyzed pigments in brown algae from the Atlantic North Coast, including *Himanthalia elongata*, *Laminaria ochroleuca*, and *Undaria pinnatifida*, though fucoxanthin levels varied between 2.79 µg/g and 26.8 µg/g [153]. Lastly, Negreanu-Pîrjol et al. (2020) examined chlorophyll pigment content in algae harvested from the Romanian coast of the Black Sea and correlated these findings with antioxidant activity [169].

#### 3.4.6. Polyphenols from Seaweeds

Phenolic compounds are secondary metabolites essential for the defense and survival of marine organisms in highly competitive environments. According to Getachew et al. (2020), macroalgae rely on phenolic compounds in their metabolic pathways to protect against environmental stress and biological threats, including against UV radiation, herbivory, and oxidative damage [170]. Jacobsen et al. (2019) identified and characterized various phenolic compounds in brown, green, and red seaweeds [171]. These marine phenolics exhibit diverse biological activities, such as anti-inflammatory effects, studied by Tenorio-Rodríguez et al. (2019), anti-cancer properties reported by Abdelhamid et al. (2019), and antioxidant and ACE inhibitory activities reported by Vijayan et al. (2018) [172,173,174].

Extraction of phenolic compounds. Traditionally, phenolic compounds have been extracted from marine sources using organic solvents, with solid–liquid extraction (SLE) being the most common method. The Soxhlet technique employs solvents such as methanol, acetone, ethanol, trichloromethane, ethyl acetate, and water-organic solvent mixtures in various ratios, as reported by Catarino et al. (2019) [175]. However, traditional methods face criticism due to their high solvent consumption, long extraction times, and high temperatures, which can lead to oxidation and hydrolysis of phenolic compounds as reported by Ojha et al. (2020) [176]. To address these limitations, alternative extraction technologies have been explored. Garcia-Vaquero et al. (2020) reviewed enzyme-assisted extraction (EAE), microwave-assisted extraction (MAE), ultrasound-assisted extraction (UAE), supercritical fluid extraction (SFE), and pressurized solvent extraction (PLE), which utilize novel solvents and milder conditions [177]. They also discussed less conventional methods like pulsed electric field (PEF)-assisted extraction and ohmic heating, which generate heat through electric currents, as well as combined extraction techniques [177]. Several studies have demonstrated the effectiveness of these methods. Habeebullah et al. (2020) used EAE to extract phenolic compounds from *Sargassum boveanum*, *Sargassum angustifolium*, and *Feldmannia irregularis* (Phaeophyceae), highlighting their antioxidant and antimicrobial properties [178]. Abdelhamid et al. (2019) used MAE on *Ericaria sedoides* (formerly *Cystoseira sedoides*) (Phaeophyceae), demonstrating anti-cancer effects, while Dang et al. (2018) applied MAE to *Sargassum vestitumsi*, and reported their antioxidant activity [173,179]. UAE was employed by Dang et al. (2018) to extract phenolics from *Hormosira banksia* (Phaeophyceae), showing antioxidant potential [179]. Týskiewicz et al. (2018) conducted an in-depth analysis of the SFE method for plant-derived phenolics, and Gallego et al. (2019) provided insights into subcritical and supercritical fluid extraction for bioactive compounds from plants, seaweeds, and microalgae [180,181]. Pangestuti et al. (2019) investigated subcritical water extraction for functional materials from the tropical red seaweed *Hypnea musciformis* (Rhodophyta) [182]. Another promising method is pressurized liquid extraction (PLE). Otero et al. (2019) demonstrated its effectiveness for extracting bioactive fatty acids and phenols from *Laminaria ochroleuca* [183]. These advancements in extraction technologies offer more sustainable and efficient approaches to harnessing the bioactive potential of marine phenolic compounds. Getachew et al. (2020) showed that the most frequently reported phenolic compounds are simple phenolic components such as gallic acid, epicatechin, epigallocatechin, and flavonoids such as myricetin, quercetin, and rutin, which are presented in [170]. Choudhary et al. (2023) reported TFC and TPC for representative algae from all three categories: green (*Ulva lactuca*, *Acrosiphonia orientalis*, and *Caulerpa scalpelliformis*), red (*Scinaia carnosa* and *Halymenia porphyriformis*), and brown (*Sargassum linearifolium* and *Iyengaria stellata*) from the Arabian Sea [53].

**Figure 8:**
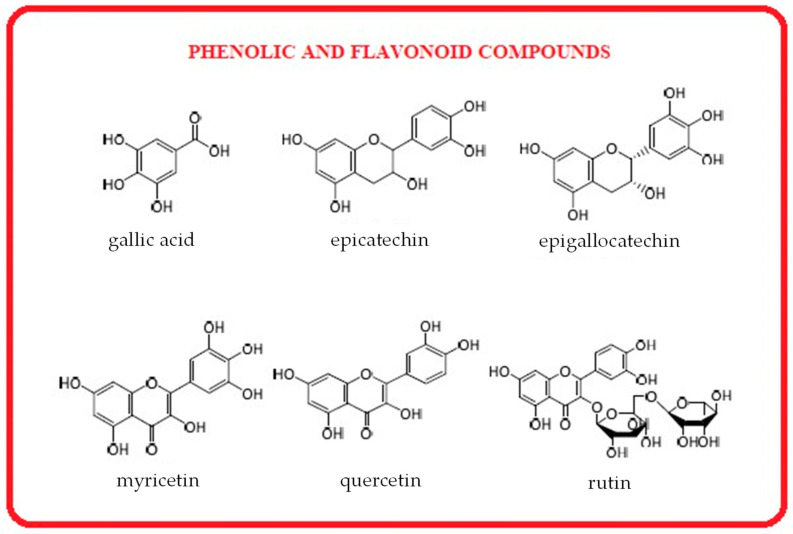
Chemical structures of some seaweed phenolic and flavonoid compounds.

For green algae, TPC and TFC values have been documented by various studies. Sirbu et al. (2020) provided data for *Cladophora vagabunda*, while Cadar et al. (2023) examined *Ulva lactuca*, *Enteromorpha intestinalis*, and *Cladophora vagabunda* [30,49]. Gentscheva et al. (2022) reported TPC and TFC values for *Ulva intestinalis*, while Wekre et al. (2019) and Dimova et al. (2019) analyzed TPC and TFC content for *Ulva rigida* [184,185,186]. Haq et al. (2019) presented TPC and TFC content for *Chaetomorpha* sp. [187]. Additionally, Sanger et al. (2019) reported TPC values for *Halimeda macroloba* [188]. Notably, Cadar et al. (2023) found the highest TPC (416.6 ± 1.56 mg GAE/100 g d.w.) and TFC (15.6 ± 1.65 mg QE/100 g d.w.) in *Ulva lactuca* harvested from the Black Sea, Romania [49]. The chemical structures of phenolic and flavonoid compounds are shown in Figure 8.

For red algae, several studies have reported TPC and TFC values. Sasadara et al. (2021) examined *Gracilaria* sp. (Bulung sangu), while Sobuj et al. (2021) analyzed *Hypnea pannosa* [189,190]. El Shafay et al. (2021) raported TPC and TFC values for *Jania rubens* and *Ellisolandia elongata* (formerly *Corallina elongata*) (Rhodophyta) and Hmani et al. (2021) for *Gracilaria gracilis* and *Asparagopsis armata* [191,192]. Studies by Farghl et al. (2021) reported TFC and TPC values for red algae *Laurencia obtusa* and *Chondrus crispus* from the Red Sea Coast [60]. Other studies by Nursid et al. (2020) for Gracilaria verrucosa, Gunathilaka et al. (2019) for Gracilaria edulis and Siangu et al. (2019) for Eucheuma denticulatum have highlighted the content of TPC and TFC polyphenols [193,194,195].

The highest TPC (176.7 ± 6.9 mg GAE/g d.w.) and TFC (173.7 ± 6.8 mg QE/g d.w.) were recorded in *Jania rubens*, as demonstrated by El Shafay et al. (2021) [191]. For brown algae, the phenolic content (TPC) was reported by by Praiboon et al. (2018) for the brown algae *Sargassum oligocystum*, by Fouda et al. (2019) for the algae *Sargassum aspirofolium* and Cadar et al. (2019) for the brown algae *Cystoseira barbata*, [61,63,196]. Iylias et al. (2023) reported TPC and TFC values for *Himanthalia elongata*, [62]. Table 6 summarizes the TPC and TFC values for representative macroalgae across all three categories—green, red, and brown algae.

Gentscheva et al. (2022) reported TPC and TFC values for the algae *Ericaria crinita* [184]. Sobuj et al. (2021) identified TPC and TFC values in *Sargassum corrifolium*, and El Shafay et al. (2021) reported results for polyphenols analyzed in *Taoria atomaria* and *Padina pavonica* [190,191]. Additionally, Subbiah et al. (2023) reported TPC and TFC values for Phyllospora comosa and *Ecklonia radiata*, while Abdelhamid et al. (2018) studied polyphenols from *Cladostephus spongiosum* that support antioxidant, anti-inflammatory, and antinociceptive potential [197,198]. The highest TPC content was reported by Cadar et al. (2019) for *Cystoseira barbata* collected from the Black Sea coast [196]. Overall, all studies confirm the presence of phenolic compounds in marine algae.

#### 3.4.7. Vitamins from Marine Macroalgae

Macroalgae are potential sources of vitamins. Bekah et al. (2023) highlighted that the global ocean provides abundant seaweeds rich in essential nutrients, particularly vitamins and minerals, which are crucial for human consumption in both water-soluble and fat-soluble forms [199]. Among the most prevalent water-soluble vitamins in seaweeds are vitamin C (ascorbic acid), B1 (thiamine), B2 (riboflavin), B3 (niacin), and B12 (cobalamin) as Lovander et al. (2018) reported [200]. Fat-soluble vitamins, such as A and E, have also been identified. Various extraction methods have been reported, such as: alcoholic and water extracts for green algae reported by Cadar et al. (2023) and soluble acid and enzymatic hydrolysis for brown algae reported by Ilyas et al. (2023) [49,62]. The determination of vitamins follows official AOAC/2019 regulations [201].

**Figure 9:**
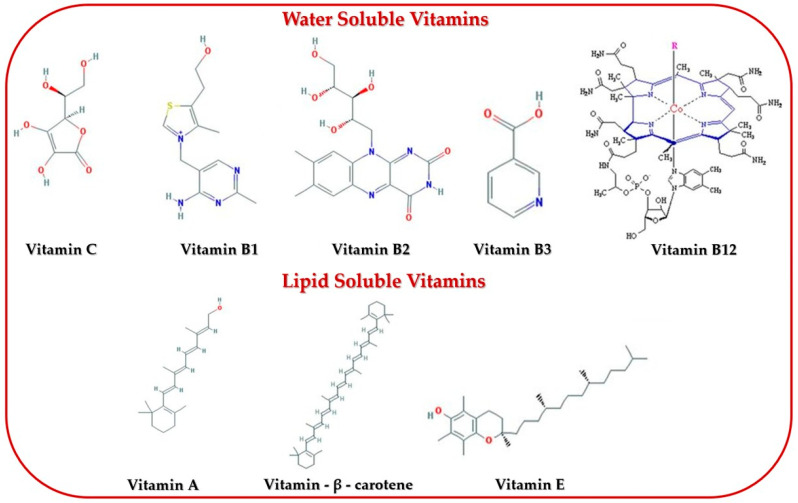
Chemical structure of the most common vitamins in seaweeds.

Figure 9 illustrates the structures of the most common vitamins found in marine macroalgae.

The following quantification techniques are highlighted as techniques for determining vitamins: UV-Vis spectrophotometry, HPLC, fluorimetry, chemiluminescence, capillary electrophoresis, and microbiology [49,62,201]. Table 7 presents values for the most representative vitamins identified in the composition of seaweeds. The analysis of the values reported for the vitamin content highlighted the existence of significant amounts of vitamin C, followed by vitamin E in almost all the seaweeds presented.

Additionally, Susanti et al. (2020) explored alternative methods, including ultrasonic-assisted extraction (UAE) of vitamin B12 (cobalamin) from the green alga *Ulva lactuca*, comparing it to traditional methods [202]. Chandra-Hioe et al. (2020) further contributed to improving UAE efficiency by implementing measures to protect cyanocobalamin from thermal degradation during extraction [203].

Bekah et al. (2023) showed the highest content of vitamin C for *Udotea argentea* harvested from the Mauritio coast as well as significant quantities for vitamins B1 and B3 [199]. High vitamin C percentages were also reported by Cadar et al. (2023) for the green alga *Cladophora vagabunda* (149.66 ± 0.58 mg/100 g. d.w.) and Metin et al. (2018) for the alga *Ulva intestinalis* (147 ± 2.00 mg/100 g. d.w.) [49,56]. Pereira et al. (2021) provided data on the fat-soluble and water-soluble vitamin content of the green alga *Ulva lactuca*, identifying vitamin B2 (0.533 mg/100 g d.w.) as the most abundant among the vitamins analyzed [204]. Several studies have also reported the vitamin composition in different algal species. Sirbu et al. (2020) and Cadar et al. (2023) analyzed the presence of vitamins A, C, E, B1, B2, and B3 in *Cladophora vagabunda*, while Morais et al. (2020) examined *Ulva rigida* for their vitamin content [30,49,54]. Ganesan et al. (2020) reported the existence of vitamins C, B2, and B3 in the composition of the green algae *Ulva lactuca* (as *Ulva fascinata)* and *Ulva flexuosa* harvested from Indian waters and vitamins A, C, E, B1, B2, and B3 were reported by Metin et al. (2018) for the algae *Ulva intestinalis* from Gulf of Gökova of Aegean Sea [55,56]. Morais et al. (2020) also documented the vitamin composition of the red algae *Palmaria palmata* and *Porphyra umbilicalis* in the Atlantic Ocean [54].

Further studies by Ganesan et al. (2020) identified vitamins C, B2, and B3 in *Gracilaria edulis* from Indian waters, while Cadar (2017) reported the vitamin content in *Ceramium virgatum* from the Black Sea [55,57]. Rosemary et al. (2019) provided data on vitamins A, C, E, B1, B2, and B3 in the red algae *Gracilaria eludis* and *Gracilaria corticata* [59]. Bekah et al. (2023) highlighted the presence of vitamins B1 and B12 in *Gracilaria corticata* from the Mauritius coast [199]. Pereira et al. (2021) also reported the vitamin content in *Chondrus crispus* from the Spanish coast [204]. Sultana et al. (2013) confirmed both fat-soluble and water-soluble vitamins in *Porphyra umbilicalis* [205]. Ryzhik et al. (2021) identified only vitamins B3 and B12 in *Palmaria palmata* from the Barents Sea [206].

Overall, red algae are rich sources of vitamins C and A. The vitamin content of brown algae has also been extensively studied. Morais et al. (2020) reported vitamins in several species from the Atlantic Ocean: *Fucus vesiculosus* (A, C, B1, B2), *Laminaria digitata* (C, E, B1, B2, B3), and *Undaria pinnatifida* (A, C, E, B1, B2, B3) [54]. Ganesan et al. (2020) identified vitamins C, B2, and B3 in *Padina gymnospora* [55]. Cadar (2017) reported vitamins C, E, B1, B2, and B3 in *Cystoseira barbata* from the Romanian Black Sea coast [57]. Ilyas et al. (2023) reported vitamins E, B1, and B2 in *Himanthalia elongata* from the North-Eastern Atlantic Ocean [62]. Bekah et al. (2023) analyzed seaweeds from the Mauritius coast, detecting vitamins B1, B2, and B3 in *Sargassum obovatum* and vitamins B1, B2, and B12 in *Padina boryana* [199]. Pereira et al. (2021) also found vitamins A, C, E, B1, and B2 in *Himanthalia elongata* from the Spanish coast [204]. Sultana et al. (2013) identified vitamins C, E, B1, B2, and B12 in *Ascophyllum nodosum* from the Irish coast [205]. In conclusion, brown seaweeds contain significant levels of vitamins C and E. Given their high vitamin content, seaweeds have potential applications as nutraceuticals and functional ingredients, contributing to their roles as antioxidants, antimicrobials, and growth factors. Furthermore, Dragomir et al. (2024) studied the impact of vitamins for pregnant women with implications on the health of the newborn for the prenatal phase and important consequences for the human body [207].

**Table 7:** Vitamin content from green, red, and brown seaweeds. Units of measurements are indicated in the first column of the table.

**Green algae**										
**Seaweed species**	***Cladophora vagabunda***	***Cladophora vagabunda***	***Ulva lactuca***	***Ulva lactuca***	***Ulva rigida***	***Ulva fasciata***	***Ulva intestinalis***	***Ulva flexuosa***	***Ulva intestinalis***	***Udotea argentea***
Region	Black Sea	Black Sea	Black Sea	Ireland coast	Atlantic waters	Indian waters	Black Sea	Indian waters	Gulf of Gökova of Aegean Sea	Mauritius coast
Vitamin Content										
Vitamin A; mg/100 g d.w.	0.151 ± 1.83	0.58 ± 0.03	0.57 ± 0.06	0.017	9581	-	0.49 ± 0.05	-	0.081 ± 1.54	-
Vitamin C; mg/100 g d.w.	89.665 ± 2.58	149.66 ± 0.58	146.63 ± 0.95	0.242	9.42	0.38 ± 0.04	136.16 ± 0.85	0.36 ± 0.02	147 ± 2.00	435
Vitamin E; mg/100 g d.w.	8.132 ± 1.03	8.54 ± 0.63	8.22 ± 0.11	0.024	19.70	-	9.93 ± 0.83	-	5.13 ± 1.03	-
Vitamin B1; mg/100 g d.w.	0.153 ± 0.02	4.16 ± 0.25	3.72 ± 0.25	-	0.47	-	3.95 ± 0.52	-	0.17 ± 0.1	48.3
Vitamin B2; mg/100 g d.w.	0.893 ± 0.16	0.89 ± 0.06	0.99 ± 0.07	0.533	0.199	0.32 ± 0.29	0.97 ± 0.02	0.26 ± 0.32	0.89 ± 0.02	-
Vitamin B3; mg/100 g d.w.; ppm *	2.495 ± 0.19	2.59 ± 0.32	2.97 ± 0.28	98 *	0.5	1.02 ± 0.41	1.84 ± 0.45	0.92 ± 0.48	2.42 ± 0.09	32.8
References	[30]	[49]	[49]	[204]	[54]	[55]	[49]	[55]	[56]	[199]
**Red algae**										
**Seaweed species**	***Palmaria palmata***	***Porphyra umbilicalis***	***Gracilaria edulis***	***Ceramium virgatum***	***Gracilaria cortica***	***Gracilaria edulis***	***Gracilaria corticata***	***Chondrus*** ***crispus***	***Porphyra umbilicalis***	***Palmaria palmata***
Region	Atlantic waters	Atlantic waters	Indian waters	Black sea	Southeast coast of India	Southeast coast of India	Mauritius coast	Spanish coast	Ireland coast	Barents Sea
Vitamin Content										
Vitamin A; mg/100 g d.w. *; mg/g **	1.59 *	3.65 *	-	-	2.67 ± 0.30 **	2.07 ± 0.06 **	-	0.1 *	3.65 *	-
Vitamin C; mg/100 g d.w. *; mg/g **; ppm ***	6.34–34.5 *	4.214 *	0.25 ± 0.06 *	50.0 ± 0.5 *	14.66 ± 0.23 **	13.41 ± 0.57 **	-	10 ***	12.885 *	-
Vitamin E; mg/100 g d.w.; mg/g **	2.2–13.9	-	-	250 ± 1.1	1.40 ± 0.10 **	1.49 ± 0.10 **	-	-	0.114	-
Vitamin B1; mg/100 g d.w.; mg/g **	0.073–1.56	0.144	-	4.2 ± 0.3	0.38 ± 0.02 **	0.36 ± 0.02 **	23.3	0.1	0.077	-
Vitamin B2; mg/100 g d.w.; mg/g **; µg/100 g ***	0.51–1.91	0.36	0.12 ± 0.15	6.6 ± 0.4	0.05 ± 0.01 **	1.54 ± 0.07 **	-	2.5	0.274	35.38 ± 2.22 ***
Vitamin B3; mg/100 g d.w.; mg/g **; µg/100 g ***	1.89	-	0.52 ± 0.28	15.0 ± 0.6	1.54 ± 0.39 **	1.10 ± 0.29 **	-	3.2	0.761	18.6 ± 1.10 ***
Vitamin B12; mg/100 g d.w.; ppm **; µg/100 g ***	-	-	-	-	-	-	26.9	0.6 **	0.769 ***	-
References	[54]	[54]	[55]	[57]	[59]	[59]	[199]	[204]	[205]	[206]
**Brown algae**										
**Seaweed species**	***Fucus vesiculosus***	***Laminaria digitata***	***Undaria*** ***pinnatifida***	***Padina gymnospora***	***Gongolaria barbata***	***Himanthalia elongata***	***Sargassum obovatum***	***Padina boryana***	***Himanthalia elongata***	***Ascophyllum nodosum***
Region	Atlantic waters	Atlantic waters	Atlantic waters	Indian waters	Black sea	North-Eastern Atlantic Ocean	Mauritius coast	Mauritius coast	Spanish coast	Ireland coast
Vitamin Content										
Vitamin A; mg/100 g d.w.	0.30–7	-	0.04–0.22	-	-	-	-	-	0.079	-
Vitamin C; mg/100 g d.w.	14.124	35.5	5.29	0.29 ± 0.02	22.0 ± 1.2	-	-	-	28.56	0.654
Vitamin E; mg/100 g d.w.; µg/g d.w. *	-	3.43	1.4–2.5	-	120 ± 1.9	33.3 ± 4.2 *	-	-	5.8	0.029
Vitamin B1; mg/100 g d.w.; g/g d.w. **	0.02	1.250	0.17–0.30	-	2.3 ± 0.5	0.14 ± 0.02 **	56.8	8.87	0.020	0.216
Vitamin B2; mg/100 g d.w.; g/g d.w. **	0.035	0.138	0.23–1.4	0.08 ± 0.18	5.5 ± 0.8	1.14 ± 0.14 **	3.27	1.67	0.020	0.058-
Vitamin B3; mg/100 g d.w.	-	61.2	2.56	0.34 ± 0.16	22.0 ± 0.9	-	17.3	-	-	-
Vitamin B12; mg/100 g d.w.; µg/100 g d.w. ***	-	-	-	-	-	-	-	24.5	-	1.840 ***
References	[54]	[54]	[54]	[55]	[57]	[62]	[199]	[199]	[204]	[205]

#### 3.4.8. Mineral Content of Seaweeds

Minerals are found in the composition of all seaweeds in different percentages. Soares, et al. (2020) showed that there are diverse criteria by which minerals are categorized according to their importance and quantity needed by plants as macronutrients: N, P, K (essential for plants in large amounts), secondary macronutrients: Ca, Mg, S (needed by plants in large doses), micronutrients: Fe, Mn, Zn, Cu, Ni, B, Mo, Cl (vital for plants in small quantities and usually toxic in high concentrations), beneficial minerals: Na, Si, Co, Al, V, Ni, Se, As, F, Br, I, Cd, Cr, Pb (essential for some plants), and potentially toxic elements: Cd, Cr, Pb, Hg, Ni, Se, As (mainly toxic to humans and animals) [208]. Choudhary et al. (2021) reported that seaweed can contain a high mineral content, sometimes comprising up to 40% of its biomass. This is due to seaweed’s ability to absorb metal ions from salt water and store them as carbonate salts [66]. Several studies, including those by Choudhary et al. (2023), de Morais et al. (2020), and Ganesan et al. (2020), have highlighted that seaweed contains significant amounts of minerals such as Na, K, Ca, and Mg, while other minerals are present in trace amounts [53,54,55]. Both conventional and modern extraction methods have been used to analyze seaweed minerals. Soares et al. (2020) employed subcritical water extraction (SWE) for extracting minerals from the brown algae *Saccorhiza polyschides*, with mineral composition analyzed via inductively coupled plasma mass spectrometry (ICP-MS) [208]. Other researchers, including Amlani et al. (2022), Choudhary et al. (2023), and Adamassu et al. (2018), have reported the use of Atomic Absorption Spectroscopy (AAS) for mineral analysis [50,53,209]. Specifically for iodine content, Choudhary et al. (2023) identified ion chromatography (IC) as an analytical method, whereas Ganesan et al. (2020) used AAS [53,61]. Brown seaweeds have the highest iodine content, while red and green seaweeds contain lower levels. Choudhary et al. (2021) further noted that seaweeds contain higher iodine levels than terrestrial plants, making them a viable dietary alternative for meeting iodine requirements compared to plant- and animal-based foods [66]. In some seaweed species, iodine concentrations exceed the daily recommended intake of 150 mg/day [66].

Ganesan et al. (2020) also found that the Na:K ratio in seaweed varies by species, ranging from 0.59 to 0.82 [55]. The highest Na content in green algae was recorded by Cadar et al. (2023) in *Ulva intestinalis* (1230.56 ± 1.65 mg/100 g d.w.) [49]. For red algae, de Morais et al. (2017) reported the highest Na concentration in *Palmaria palmata* (1600–2500 mg/100 g d.w.) [54], while in brown algae, Choudhary et al. (2023) recorded the highest Na level in *Iyengaria stellata* (11,000 ± 250 mg/100 g d.w.) [53].

Potassium (K), an essential element for human health, was found in the highest concentration in green algae *Caulerpa scalpelliformis* (9300 ± 250 mg/100 g d.w.) and in brown algae *Iyengaria stellata* (117,000 ± 400 mg/100 g d.w.), as reported by Choudhary et al. (2023) [53]. The highest K content in red algae was reported by de Morais et al. (2020) in *Palmaria palmata* (7000–9000 mg/100 g d.w.) [54].

Calcium (Ca), a secondary macronutrient, was found in the highest concentration in green algae *Ulva lactuca* (1790.35 ± 2.55 mg/100 g d.w.), as identified by Cadar et al. (2023) [49]. The highest Ca content in red algae was recorded in *Laurencia obtusa* (845.35 ± 0.11 mg/100 g d.w.) by Farghl et al. (2021) [60], while in brown algae, Ilyas et al. (2023) reported the highest level in *Himanthalia elongata* (3469 ± 1526 mg/100 g d.w.) [62].

In Table 8 are presented values for the mineral content reported by different authors in the composition of marine seaweeds: green, red, and brown.

For magnesium (Mg), Choudhary et al. (2023) recorded the highest concentration in green algae *Acrosiphonia orientalis* (1400 ± 100 mg/100 g d.w.) and in red algae *Scinaia carnosa* (4000 ± 80 mg/100 g d.w.) [53]. In brown algae, Ilyas et al. (2023) reported the highest Mg content in *Himanthalia elongata* (3537 ± 1497 mg/100 g d.w.) [62]. Cadar et al. (2023) reported the highest iron (Fe) content in green algae, specifically in *Cladophora vagabunda* (565.35 ± 1.05 mg/100 g d.w.) [49]. For red algae, Rosemary identified the highest Fe content in *Gracilaria corticata* (107.24 ± 20.9 mg/100 g d.w.), while Praiboon et al. (2018) recorded the highest Fe level in brown algae, specifically *Sargassum oligocystum* (416.92 ± 4.24 mg/100 g d.w.) [59,61]. The Fe content in seaweeds varies significantly, ranging from the highest value of 565.35 ± 1.05 mg/100 g d.w. (Cadar et al., 2023) to as low as 0.3 ± 0.01 mg/100 g d.w., observed in the brown alga *Sargassum linearifolium* by Choudhary et al. (2023) [49,53]. Ganesan et al. (2020) emphasized that seaweed-derived iron could serve as a complementary and viable source to meet physiological iron requirements, particularly for pregnant women [55]. Regarding zinc (Zn), Ganesan et al. (2020) reported the highest Zn content in green algae, specifically *Ulva flexuosa* (1.518 ± 0.81 µg/100 g d.w.) [55]. In red algae, Premarathana et al. (2022) found the highest Zn content in *Jania adhaereus* (70.94 mg/100 g d.w.), while Praiboon et al. (2018) recorded the highest Zn levels in the brown alga *Sargassum oligocystum* (21.84 ± 4.04 mg/100 g d.w.) [58,61]. Zn content in seaweeds ranges from 1.518 ± 0.81 µg/100 g d.w. in *Ulva flexuosa* (Ganesan et al., 2020) to 70.94 mg/100 g d.w. in *Jania pedunculata* var. *adhaerens* (Premarathana et al., 2022) [55,58]. Choudhary et al. (2023) found that seaweeds contain higher levels of essential microelements such as sodium (Na), potassium (K), calcium (Ca), and magnesium (Mg) compared to terrestrial vegetables like spinach, potatoes, carrots, and tomatoes [53]. Additionally, Premarathana et al. (2022) and Lozano Muñoz et al. (2022) provided comprehensive reports on seaweed nutrition, highlighting the crucial role of minerals in hormone and enzyme synthesis, as well as the importance of trace elements in disease prevention and healing [58,210].

Heavy metals, including mercury (Hg), arsenic (As), cadmium (Cd), lead (Pb), and copper (Cu), have been detected in seaweed compositions. Ganesan et al. (2020) reported Hg levels of 0.031 ppm in *A. specifera* [55]. Arsenic has been detected in several seaweed species, but only in very low concentrations (expressed in ppm). Díaz et al. (2012) reported studies on the arsenic content in different species of seaweeds collected from the Chilean marine coast [211]. They showed that the levels of inorganic arsenic in the studied seaweeds ranged from 0.8% to 13% of the total arsenic concentrations, so the arsenic present in the studied seaweeds is in the form of organic arsenic; in addition, the inorganic arsenic concentrations are relatively low compared to the total arsenic found in seaweeds and do not represent a major health risk to consumers [211]. Pb and Cu have also been identified in various seaweeds. According to Ganesan et al. (2020), regulatory limits for heavy metals in edible seaweeds are established, with Pb restricted to < 0.5 mg/kg d.w., Hg to < 0.1 mg/kg d.w., and inorganic As to < 3 mg/kg d.w. [55]. Studies by Cadar et al. (2019), Ganesan et al. (2020), Lozano Muñoz et al. (2022), and Díaz et al. (2012) confirmed that while heavy metals are present in seaweeds, they remain within the toxicological safety limits established by food safety authorities [9,55,210,211].

## 4. The Relationship Between the Biological Activities of Biocompounds—Potential Health and Nutraceutical Application

In the context of harnessing the benefits of utilizing biocompounds from natural resources in the treatment of various diseases as compared to traditional chemically synthesized drugs, medical and nutraceutical applications of seaweeds based on the biological actions of their biocompounds have been considerably increased. Biocompounds in seaweed compositions present important biological actions that have been studied by multiple researchers for different medical and nutraceutical applications, such as those presented by Ahmed N. et al. (2024), Xu et al. (2023), and Silva M. et al. (2024) [212,213,214].

**Figure 10:**
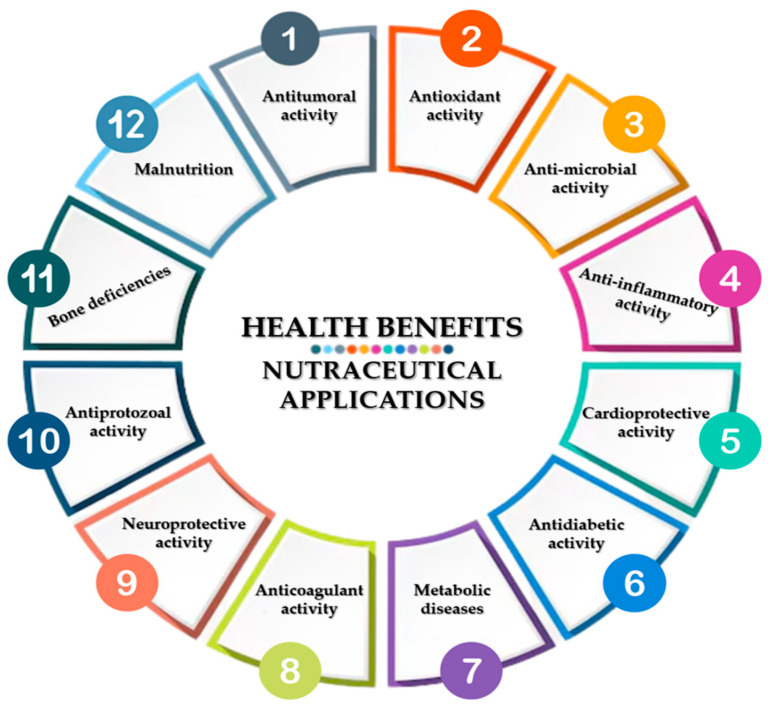
Diseases treated with nutraceuticals containing marine algae biocompounds with various biological activities.

Figure 10 systematizes the biomedical applications of marine algae biocompounds for treating various conditions.

### 4.1. Antitumoral Activity

Cancer is a major public health problem and the second leading cause of death worldwide after cardiovascular disease as shown by Ouyang et al. (2021) [16]. It is important for health that alternative treatments can be used for certain diseases such as cancer. Antitumor activity has been reported for various active metabolites from seaweed such as polysaccharides, terpenoids, pigments, and polyphenols, as argued by various studies.

Several studies have been reported in which polysaccharides exhibit multiple anti-cancer activities. Thus, the antitumor activity of fucoidans was reported by Mabate et al. (2023) in studies on three brown algae, *Fucus vesiculosus*, *Ecklonia radiata*, and *Sargassum elegans* [215]. Shiau et al. (2022), in the research carried out on *Fucus vesiculosus*—brown algae—showed that fucoidans have the activity of reducing colony formation, cancer cell formation, and cell adhesion [216]. Cao et al. (2022) indicate the antitumor activity of fucoidans in studies conducted on *Ulva conglobate*—green algae—by the induction of apoptosis and cell cycle reduction in HT-29 cells in the S and G2/M phase and accumulation in G1 phase cells [217]. Gao et al. (2021) demonstrated that fucoidans from *Sargassum pallidum* have antitumoral and immune-enhancing activities [218]. Bellan et al. (2020) showed that biocompounds with sulphated galactan structures from *Codium isthmocladum*—green algae—exhibited reduced cell invasion, colony-forming capacity, and reduced solid tumour growth and metastasis [219]. Examples of studies that have shown antitumoral activity of biocompounds from seaweed are presented in Table 9.

Zhao et al. (2020) discovered antitumoural activities of polysaccharides from *Ulva Lactuca*—green algae—manifested by decreasing dicarboxylic aldehyde methane dicarboxylic acid levels and inhibiting the activation of signaling pathways in human uveal melanoma cells [220]. Yao et al. (2020) reported that porphyrans from *Pyropia haitanensis* (formerly *Porphyra haitanensis*)—red algae—exhibited direct cytotoxic effects, inducing oxidative stress and apoptosis in cells and causing G0-G1 phase arrest of cells [72]. Cicinskas et al. (2020) demonstrated the antitumoral activities of carrageenans from *Chondrus armatus*—red algae—by reducing the cell viability of cancer cells, and Choi et al. (2019) reported that sulfated glucuronorhamnoxylan from *Capsosiphon fulvescens*—green algae—inhibits the growth of human colon cancer cells HT-29 [221,222]. Mendes Marques et al. (2019) studied sulfated galactans from *Udotea flabellum*—green algae—and showed that the anti-proliferative activity was dependent on their degree of sulfation, and Narayani et al. (2019) reported that polysaccharides from *Sargassum cinereum*—brown algae—produced a reduction in the cell viability of cancer cells [223,224].

Other classes of biocompounds have also shown anti-cancer activities. Bharathi et al. (2021) showed anti-cancer effects of SiO2–ZnO nanocomposites with diterpenes from *Dictyota bartayresiana*—brown algae—and developed a drug with antitumoral activity against a cell line of colon cancer [225]. From the class of pigments, fucoxanthin from *Undaria pinnatifida*—brown algae—reported by Wang et al. (2019), produced a decrease in the level of vascular endothelial growth factor (VEGF)-C, VEGF-3 receptor, nuclear factor kappa β, phospho-Akt, and phospho-PI3K in HLEC [226]. They showed that fucoxanthin decreases microlymphatic vascular density in a nude mouse model of MDA-MB-231 breast cancer [226].

Polyphenols also showed antitumoral activities. Thus, Mahendran et al. (2024) indicated that polyphenolic compounds from *Sargassum tenerrimum*—brown algae—by MTT assay show antitumor activity against HeLa cells [227]. In alternative treatments for drugs obtained by chemical synthesis, Cadar et al. (2023) showed that biocompounds from other natural resources can be successfully used in the treatment of cancer diseases [228].

### 4.2. Antioxidant Activity

Antioxidant activity is the most widely encountered biological activity of marine algae biocompounds. Table 9 systematizes examples of antioxidant and anticoagulant activities corresponding to the classes of biocompounds that constitute active metabolites in marine macroalgae. Antioxidant activity is manifested by the following classes of compounds in seaweed: polysaccharides, terpenoids, pigments, polyphenols, and vitamins. Antioxidant activity was exhibited by polysaccharides mostly for brown algae as reported by Alboofetileh et al. (2022) for the brown algae *Nizamuddinia zanardinii* and Wang et al. (2020 a) for the brown algae *Sargassum fusiforme* (formerly *Hizikia fusiforme*) [229,230]. Oh et al. (2020) demonstrated antioxidant activity exhibited by polysaccharides from *Undaria pinnatifida*—brown algae [71]. The antioxidant activity of polysaccharides was shown by Wang et al. (2020 b) from *Ecklonia maxima* and Jayawardena et al. (2020) from *Padina boryana*, respectively [231,232]. Le et al. (2019) reported the antioxidant activity of ulvan for the green alga *Ulva lactuca* (as *Ulva pertusa*) [233]. Wang et al. (2019) and Maneesh et al. (2018) reported the antioxidant activity of polysaccharides also from brown algae: *Sargassum fulvellum* and *Sargassum wightii*, respectively [234,235]. Yang et al. (2021) evidenced the protective actions of polysaccharide extracts from *Ulva lactuca*, which suppresses kidney damage and decreases oxidative stress in the kidneys [236]. The antioxidant activity due to terpenoids in the composition of algae was reported by Zhang et al. (2020) from the red algae *Laurencia tristicha* [237]. The antioxidant activity of fatty acids was evaluated from *Palmaria palmata*—red algae—by Lopes et al. (2019), and from *Grateloupia turuturu*—red algae—by Da Costa et al. (2021) [126,238]. The antioxidant activity due to proteins and protein compounds in the composition of algae was reported by Echave et al. (2022) for representatives of brown, green, and red algae, by Zhang et al. (2019) in the red algae *Gracilariopsis lemaneiformis* [135,239], Torres et al. (2019) and Torres et al. (2018) in the red algae *Pyropia yezoensis* and other red algae from the phylum *Rodophyta*—*Porphyra* spp. [240,241]. The antioxidant actions of peptides from other marine resources such as those due to collagen from marine organisms were reported by Cadar et al. in 2023 and 2024 [242,243]. Pigments constitute a category of active biocompounds, secondary metabolites that have intense anti-oxidant activity. Thus, Cadar et al. (2023) reported the antioxidant activity of chlorophyll pigments and total carotenoids from the green algae *Ulva lactuca*, *Ulva intestinalis*, and *Cladophora vagabunda* [49]. Sudhakar et al. (2023) reported the antioxidant activity of the pigments from *Gracilaria corticate*—red algae [244]. Yalçın et al. (2021) indicate the antioxidant activity of chlorophyll pigments, carotenoids, and fucoxanthin, respectively, from the green algae *Caulerma racemosa*, *Hypnea musciformis*—red algae—and from *Cladostephus spongiosus*—brown algae [245]. Radman et al. (2021) reported the antioxidant activity of pigments from the green alga *Codium adhaeresn*, Ulagesan et al. (2021) from the red alga *Pyropia Yezoensis*, and Jerković et al. (2021) from the brown alga *Fucus virsoides* [246,247,248]. We thus find that pigments with important antioxidant activity were identified in representative algae from all green, red, and brown taxa. Negreanu-Pîrjol et al. (2020, 2021) studied the antioxidant activity of pigments from red and green algae from the Black Sea basin, *Ceramium virgatum*, and *Ulva lactuca* [169,249]. The antioxidant activity due to fucoxanthin from brown algae was reported by Karkhaneh et al. (2020) from the alga *Dictyota cervicornis* (formerly *Dictyota indica*) [250]. Ghaliaoui et al. (2020) showed the antioxidant activity of pigments from *Phyllariopsis brevipes* (formerly *Phyllaria reniformis*) (Phaeophyceae) [251]. Mohibbullah et al. (2018) showed the neuroprotective effects of fucoxanthin from *Undaria pinnatifida* in attenuating oxidative stress in hippocampal neurons [252]. Fu et al. (2024) explained through scientific arguments the relationship between the structure of polysaccharide biocompounds from macroalgae and biological actions in curing various diseases such as antioxidant, anti-inflammatory, anticoagulant, antiviral, immunomodulatory, and antitumoral activities [253]. Polyphenols constitute another valuable category of compounds that have antioxidant activity. For brown algae, Mahendran et al. (2024) reported the antioxidant activity of polyphenols from *Sargassum tenerrimum*, [227]. Generalić Mekinić et al. (2021) reported the antioxidant activity of polyphenols from *Dictyota dichotoma* and from *Padina pavonica*—brown algae—and Hassan et al. (2021) reported the antioxidant activity of polyphenols from *Padina boryana*—brown algae—and *Acanthophora spicifera*—red algae [254,255]. The antioxidant activity of phenolic compounds from *Gongolaria barbata*—brown algae—was revealed by Cadar et al. (2019) and from *Chondrus crispus* species—red algae—by Alkhalaf et al. (2021) [196,256]. The antioxidant activity of flavonoid compounds was reported by Ak et al. (2018) from *Gongolaria barbata*—brown algae—and *Gigartina acicularis*—red algae [257]. Neuroprotective actions due to polyphenolic compounds from macroalgae have also been presented by Alghazwi et al. (2020), Shrestha et al. (2020), and Yang et al. (2018) from various brown algae [258,259,260]. Vitamins from the composition of marine algae also have antioxidant activity. Thus, Le et al. (2024) studied the antioxidant activity of vitamins from *Odonthalia dentata*—red algae [261]. In particular, vitamin C shows antioxidant activity as reported by Subramoni 2023 for *Caulerpa chemnitzia*—green algae [262]. Methods for demonstrating antioxidant activity are ABTS, Frap, and CUPRAC assay. Antioxidant activity decreases intracellular ROS and lipid peroxidation, increases the body’s immunity, protects against oxidative stress, and participates in metabolic regulatory mechanisms.

**Table 9:** Biological compounds of seaweeds with antitumoral and antioxidant activity.

Type of Seaweed	Bioactive Metabolites/ Compounds	Mechanism of Action	Biological Activity	References
**Antitumoral activity**				
Polysaccharides				
*Fucus vesiculosus*—brown algae	Fucoidan	Decreases colony formation, cancer cell formation, and cell adhesion.	Antitumoral activity	[215]
*Ecklonia radiata*—brown algae	Fucoidan	Decreases cancer cell formation and cell adhesion.	Antitumoral activity	[215]
*Sargassum elegans*—brown algae	Fucoidan	Decreases colony formation, cancer cell formation, and cell adhesion.	Antitumoral activity	[215]
*Fucus vesiculosu*—brown algae	Fucoidans	Decreases cancer cell sphere formation and cell adhesion.	Antitumoral activity	[216]
*Ulva conglobata*—green algae	Fucoidans	Induced apoptosis and decreased the cell cycle in HT-29 cells in S and G2/M phases and accumulation in cells in G1 phase. It has been proven that H_2_O_2_ has an antioxidant effect on HT-29 cells.	Antitumoral activity	[217]
*Sargassum pallidum*—brown algae	Fucoidans	Decreases colony formation, cancer cell sphere formation, and cell adhesion.	Antitumoral activity	[218]
*Codium isthmocladum*—green algae	Sulphated galactan	Reducing cell invasion, colony-forming capacity, and membrane glycoconjugates and reducing solid tumour growth and metastasis.	Antitumor activity	[219]
*Ulva Lactuca*—green algae	Polysaccharides	Decreased the level of methane dicarboxylic aldehyde and inhibited the activation of signaling pathways in human uveal melanoma cells	Antitumor activity	[220]
*Pyopia haitanensis*—red algae	Porphyrans	Direct cytotoxic effects, inducing the oxidative stress and apoptosis in cells, and causing the cell G0–G1 phase arrest.	Antitumoral activity	[73]
*Chondrus armatus*—red algae	Carrageenans	Decreases cell viability of cancerous cells.	Antitumoral activity	[221]
*Capsosiphon fulvescens*—green algae	Sulfated glucuronorhamnoxylan	Inhibits the growth of HT-29 human colon cancer cells.	Antitumor activity	[222]
*Udotea flabellum*—green algae	Sulfated galactans	The anti-proliferative activity was dependent on their degree of sulfation.	Antitumoral activity	[223]
*Sargassum cinereum*—brown algae	Polysaccharides	Decreases cell viability of cancerous cells.	Antitumoral activity	[224]
Terpenoides				
*Dictyota bartayresiana*—brown algae	SiO_2_–ZnO nanocomposites with diterpenes from algae	Antitumor effect on HT29. Antimicrobian effect. Excellent antioxidant activity.	Antitumor potential	[225]
Pigments				
*Undaria pinnatifida*—brown algae	Fucoxanthin	Decreases levels of vascular endothelial growth factor (VEGF)-C, VEGF receptor-3, nuclear factor kappa β, phospho-Akt, and phospho-PI3K in HLEC. Decreases micro-lymphatic vascular density in an MDA-MB-231 nude mouse model of breast cancer.	Antitumoral activity on breast cancer.	[226]
Polyphenols				
*Sargassum tenerrimum*—brown algae	Polyphenol compounds	Polyphenols have anti-cancer activity against HeLa cells.	Antitumoral activity	[227]
**Antioxidant activity**				
Polysaccharides				
*Nizamuddinia zanardinii*—brown algae	Fucoidan	It decreases the intracellular production of ROS, having a protective effect	Antioxidant activity	[229]
*Sargassum fusiforme* (as *Hizikia fusiforme*)—brown algae	Fucoidan	It reduced apoptosis by eliminating intracellular ROS, by increasing intracellular SOD-1 and CAT expressed by up-regulation of Nrf2. Prevented cell death.	Antioxidant activity	[230]
*Undaria pinnatifida*—brown algae	Fucoidan	Decreases cell death, intracellular ROS, and lipid peroxidation	Antioxidant activity	[71]
*Ecklonia maxima*—brown algae	Sulfated polysaccharides	It decreases oxidative stress and cell death, improves inhibition of MMPs.	Antioxidant, activity	[231]
*Padina boryana*—brown algae	Sulfated polysaccharides	Decreases cell death, intracellular ROS, and lipid peroxidation	Antioxidant activity	[232]
*Ulva australis* (as *Ulva pertusa*)—green algae	Ulvan	Showed antioxidant activity by increasing antioxidant enzymes CAT, SOD, GPx	Antioxidant activity	[233]
*Sargassum fulvellum*—brown algae	Polysaccharides	Decreases cell death, decreases intracellular ROS and lipid peroxidation	Antioxidant activity	[234]
*Sargassum wightii*—brown algae	Sulfated polygalacto-pyranosyl-fucopyranan	The presence of sulphate groups in the composition of the isolated polysaccharide seems to play a major role in the scavenging potential of free radicals.	Antioxidant activity	[235]
*Ulva lactuca*—green algae	Polysaccharides	protective effect on renal lesions by decreasing atrophy and serum levels of creatinine and cystatin C. Decreases oxidative stress in the kidneys.	Kidney injury caused by oxidative stress	[236]
Terpenoides				
*Laurencia tristicha*—red algae	Laurane-type sesquiterpene	Different methods of evaluating antioxidant activity	Antioxidant activity	[237]
Fatty acids				
*Grateloupia turuturu*—red algae	EPA and PUFA	Free radical scavenging activity: DPPH and ABTS	Antioxidant activity	[238]
*Palmaria palmata*—red algae	EPA	Antioxidant assay by DPPH and ABTS	Antioxidant activity	[126]
Proteins				
Red, brown, and green seaweeds	Proteins, peptides, lectins	For antioxidant activity of amino acids: Test DPPH and ABTS assay	Antioxidant activity	[136]
*Gracilariopsis lemaneiformis*—red algae	Peptide sequence ELWKTF	Scavenging DPPH free radicals assay	Antioxidant activity	[239]
*Pyropia yezoensis*—red algae	Amino acid: Taurine	Different methods of evaluating antioxidant activity	Antioxidant activty	[240]
*Rodophyta–Porfira* spp.	Amino acids inred algae	Evaluate antioxidant capacity—DPPH, ferrous ion-chelating, ABTS, FRAP, β-carotene/linoleic acid, and ORAC	Antioxidant activity	[241]
Pigments				
*Ulva lactuca*—green algae	Chlorophyll a and b; total carotenoids	Antioxidants activity by DPPH, FRAP, TEAC assay	Antioxidant activity	[49]
*Ulva intestinalis*—green algae	Chlorophyll a and b; total carotenoids	Antioxidants activity by DPPH, FRAP, TEAC assay	Antioxidant activity	[49]
*Cladophora vagabunda*—green algae	Chlorophyll a and b; total carotenoids	Antioxidants activity by DPPH, FRAP, TEAC assay	Antioxidant activity	[49]
*Gracilaria corticata*—red algae	R-phycoerythrin	Resulted activity by MTT assay, and the colon cancer cell lines SW620 and HCT-116 were inhibited by the compound in a concentration-dependent manner.	Antioxidant activity	[241]
*Caulerma racemosa*—green algae	Chlorophyll a and b, β-carotene	Showed antioxidant activity by CUPRAC and ABTS assays.	Antioxidant activity	[245]
*Hypnea musciformis*—red algae	Fucoxanthin, chlorophyll a and b, β-carotene	Showed antioxidant activity by CUPRAC and ABTS assays.	Antioxidant activity	[245]
*Cladostephus spongiosus*—brown algae	Fucoxanthin, pheophytin-α, chlorophyll a	Showed antioxidant activity by CUPRAC and ABTS assays.	Antioxidant activity	[245]
*Codium adhaerens*—green algae	Fucoxanthin, pheophytin-α	Showed antioxidant activity by FRAP, DPPH, and ABTS assays.	Antioxidant activity	[246]
*Pyropia yezoensis*—red algae	R-phycoerythrin	Antioxidant activity by ABTS and FRAP assays and had significant cytotoxicity against Hep G2 cells	Antioxidant activity Antitumoral activity	[247]
*Fucus virsoides*—brown algae	Fucoxanthin, pheophytin-α	Proapoptotic activity for human cervical adenocarcinoma HeLa cells.	Antioxidant activity	[248]
*Ceramium virgatum*—red algae	Carotenoids, xanthophyll and β-carotene	Showed antioxidant activity by TEAC assay	Antioxidant activity	[249]
*Ulva lactuca*—green algae	Chlorophyll a and b	Showed antioxidant activity by TEAC assay	Antioxidant activity	[169]
*Dictyota cervicornis* (formerly *Dictyota indica*)—brown algae	Fucoxanthin	Strong antioxidant activity by FRAP assay	Antioxidant activity	[250]
*Phyllariopsis brevipes* (formerly *Phyllaria reniformis*)—brown algae	Fucoxanthin, pheophytin-α,	Antioxidant activity by DPPH assay	Antioxidant activity	[251]
*Undaria pinnatifida*—brown algae	Fucoxanthin	Showed protection from neurite breakage	Neurodegenerative diseases	[252]
Polyphenols				
*Sargassum tenerrimum*—brown algae	Polyphenol compound	Showed potential activity by TEAC, FRAP, H_2_O_2_, DPPH, and ABTS assays	Antioxidant activity	[227]
*Dictyota dichotoma*—brown algae	Protocatechuic, *p*-hydroxybenzoic, coumaric, and ferulic acid	Showed activity by FRAP, DPPH, ORAC assays	Antioxidant activity	[254]
*Padina pavonica*—brown algae	Protocatechuic, ferulic, *p*-hydroxy-benzoic acid	Showed activity by FRAP, DPPH, ORAC assays	Antioxidant activity	[254]
*Padina boryana*—brown algae	Polyphenolic compound	DPPH and FRAP assay	Antioxidant activity	[255]
*Acanthophora spicifera*—red algae	Polyphenolic compound: Velutin	DPPH and FRAP assay	Antioxidant activity	[255]
*Gongolaria barbata*—brown algae	Total phenolic content	DPPH radical scavenging activity and reducing power	Antioxidant activity	[256]
*Gongolaria barbata*—brown algae	Flavonoids	DPPH assay	Antioxidant activity	[257]
*Gigartina acicularis*—red algae	Flavonoids	DPPH assay	Antioxidant activity	[257]
*Ecklonia radiata*—brown algae	Phlorotannim	Inhibition of apoptosis induced by Aβ1–42	Neuroprotective activity	[258]
*Ecklonia radiata*—brown algae	Eckol-type phlorotannins	Showed neuroprotective activity against the neurotoxic amyloid β-protein (Aβ1–42) in a neuronal PC-12 cell line in vitro experiment.	Neuroprotective activity	[259]
*Eklonia cava*—brown algae	Phloroglucinol	Decreases the amyloid β-peptide burden and pro-inflammatory cytokines in the hippocampus.	Neurodogenerative disease	[260]
Vitamins				
*Odonthalia dentata*—red algae	A, B1, B2, B3, B6, B9, C, and E	Increases the body’s immunity. Protects against oxidative stress. Involved in metabolic regulation processes.	Antioxidant activity	[261]
*Caulerpa chemnitzia*—green algae	Vitamin C	Strengthening the immune system. Involvement in the cell regeneration process	Antioxidant activity	[262]

### 4.3. Antimicrobial Activity

Antimicrobial activity has been identified in the following classes of biocompounds from seaweeds as shown in Table 10: terpenoids, pigments, and polyphenols. Rushdi et al. (2022) showed that diterpenes such as dictyols from *Dictyota dichotoma*—brown algae—have antimicrobial activities on the murine macrophage cell line RAW 264.7 [114]. Sumayya et al. (2020) studied the antimicrobial activity against *Streptococcus mutans*, using purified terpenoid fractions from red algae: *Gracillaria dura*, *Hypnea musciformis*, and *Kappapycus alvarezii* algae [263]. For the evaluation of antimicrobial activity, Kuete et al. (2010) considered MIC (Minimum inhibitory concentration (MIC)) as the optimal evaluation parameter and set the antimicrobial activity parameters as follows: for extracts, the criteria were significant (MIC < 100 µg/mL), moderate (100 < MIC ≤ 625 µg/mL), or weak (MIC > 625 µg/mL) and for compounds, these stringent criteria were significant (MIC < 10 µg/mL), moderate (10 < MIC ≤ 100 µg/mL), and weak or negligible (MIC > 100 µg/mL) [264]. Sumayya et al. (2020) showed that the MIC values for terpenoid extracts purified from *Gracillaria dura*, *Hypnea musciformis*, and *Kappapycus alvarezii* against *S. mutans* were 65, 500, and 1. 500 µg/mL, respectively [263]. Also, Tamokou et al. (2017) established criteria for edible plant extracts or their parts and they were estimated to be highly active if MIC values < 100 µg/mL, significantly active if 100 ≤ MIC ≤ 512 µg/mL, moderately active if 512 ≤ MIC ≤ 2048 µg/mL, and not highly active if MIC > 2048 µg/mL [265]. The results of the MIC value study showed that purified terpenoid extracts from *Gracilaria dura* (65 μg/mL) had significant antimicrobial activity against *S. mutans*, (while weak against *E. faecalis*, *P. aeruginosa*, and *K. pneumoniae*), and *Hypnea musciformis* (MIC 500 μg/mL) showed moderate action against *S. mutans*, taking into consideration the criteria established by Kuete et al. (2010) and Tamokou et al. (2017) [263,264,265]. Ríos et al. (2005) have shown that the presence of antimicrobial activity is very interesting at concentrations below 100 µg/mL for extracts and 10 µg/mL for isolated compounds [266]. MIC 65 µg/mL for purified terpenoid extracts from *Gracillaria dura* also meets the criteria set by Rios et al. (2005), showing intense antimicrobial activity against *S. mutans* [263,266].

Anjali et al. (2019) studied the antimicrobial activities (by diffusion) against *E. coli*, *K. pneumonia*, and *S. typhi* due to sesquiterpenoids from *Ulva lactuca*—green algae [267]. Da Graça Pedrosa de Macena et al. (2023) studied the anti-Herpes simplex virus type 2 (HSV-2) activities of terpenoids from two brown algae, *Stypopodium zonale* and *Canistrocarpus cervicornis* [268]. Cirne-Santos et al. (2020) showed antiviral activity on *Zika* and *Chikungunya* viruses due to *Dolastane*-type diterpenoids from *Canistrocarpus cervicornis*—brown algae [269]. Pigments were studied by Oliyaei et al. (2021), who reported antimicrobial activity against *S. aureus* due to fucoxanthin from *Sargassum angustifolium* and *Cystoseira indica* brown algae [270]. Generalić Mekinić et al. (2021) reported the antimicrobial activities of phenolic compounds from the brown algae *Padina pavonica* (Dictyotaceae) against *B. subtilis*, *P. aeruginosa*, *S. aureus*, and *C. albicans* [254].

### 4.4. Anti-Inflammatory Activity

Biocompounds with anti-inflammatory properties include polysaccharides, fatty acids, and pigments; see Table 10. Fucoidans derived from brown algae have demonstrated anti-inflammatory activity, as reported by Liyanage et al. (2023) from *Sargassum autumnale*, Jayasinghe et al. (2023) from *Sargassum siliquastrum*, and Jayasinghe et al. (2022) from *Sargassum confusum* [271,272,273]. Other researchers have also reported anti-inflammatory activity due to seaweed biocompounds, such as Apostolova et al. (2022) from *Cystoseira crinita*, and Jayawardena et al. (2020) from *Sargassum swartzii* [274,275]. This activity is generally attributed to the inhibition of inflammatory mediators and pro-inflammatory cytokines. The anti-inflammatory effects of sulfated polysaccharides have also been documented. Wang L et al. (2022) studied sulfated polysaccharides from *Codium fragile*, while Chen et al. (2021) focused on those from *Saccharina japonica* [276,277]. In addition, other studies were conducted by Je et al. (2021) on *Sargassum binderi*, Wang L et al. (2021) on *Sargassum fulvellum*, and Wang S. et al. (2020) on *Saccharina japonica* [278,279,280]. These compounds exert anti-inflammatory effects by reducing cell death while modulating nitric oxide (NO) and reactive oxygen species (ROS) generation. Fatty acids have also been shown to possess anti-inflammatory properties. Jaworovska et al. (2023) identified this activity in saturated fatty acids (SAs) and eicosapentaenoic acid (EPA) derived from *Fucus spiralis* and *Undaria pinnatifida* [120]. Similarly, Foseid et al. (2020) reported anti-inflammatory effects from *Palmaria palmata* (red algae), while Rocha et al. (2021) found evidence of these properties in *Undaria pinnatifida* (brown algae) and *Gracilaria gracilis* (red algae) [117,118]. Pereira et al. (2021) and Berneira et al. (2020) further highlighted the role of fatty acids from marine macroalgae, particularly SFA, MUFA, and PUFA, in modulating inflammation [281,282]. Finally, pigments such as fucoxanthin, pheophytin-α, chlorophyll-a, and β-carotene have demonstrated anti-inflammatory activity. Dai et al. (2021) investigated these pigments in *Sargassum fusiforme* (brown algae) and found that they inhibit prostaglandin E2 (PGE2), cyclooxygenase-2 (COX-2), and the production of interleukins (IL)-1β and IL-6 in HaCaT keratinocytes [283].

### 4.5. Cardioprotective and ACE Inhibitory Activity

Cardiovascular diseases significantly impact human health, driving interest in natural biocompounds with cardioprotective properties. In recent years, there has been growing attention on natural ACE inhibitory peptides derived from these biocompounds, as highlighted by Cadar et al. (2024) [243]. Table 10 presents various biocompounds from marine macroalgae that exhibit both cardioprotective and ACE inhibitory activity. Marine algae contain several classes of bioactive compounds, such as polysaccharides and fatty acids, known for their cardioprotective effects. Maneesh et al. (2018) demonstrated that sulfated poly-galactopyranosyl-fucopyranan compounds from *Sargassum wightii* (brown algae) possess antihypertensive activity [235]. Similarly, Cheng et al. (2020) reported that fucoidans from *Fucus vesiculosus* (brown algae) exhibit cardioprotective properties [284]. Fatty acids from seaweeds have also shown significant potential in cardiovascular health. Polyphenols from *Palmaria palmata* (red algae) and *Alaria esculenta* (brown algae) were identified by Foseid et al. (2020) as having potential activity against coronary heart disease [117]. Furthermore, Rocha et al. (2021) highlighted the cardioprotective properties of fatty acids from *Undaria pinnatifida* (brown algae) and *Gracilaria gracilis* (red algae) [118]. Pereira et al. (2021) and Berneira et al. (2020) explored the cardioprotective effects of fatty acids—including SFA, MUFA, and PUFA—derived from *Ulva lactuca* (green algae), *Ulva intestinalis* (green algae), particularly through COX-2 enzyme inhibition mechanisms [281,282]. ACE inhibitory activity has been demonstrated in proteins and pigments from marine algae. Kumagai et al. (2021) investigated ACE inhibitory properties in 42 peptide preparations from *Pyropia pseudolinearis*, selecting ARY, YLR, and LRM peptides for further study. Their results indicated that LRM had the lowest IC50 value (0.15 mol), compared to ARY (1.3 mol) and YLR (5.8 mol) [285]. Proteins from macroalgae also exhibit strong ACE inhibitory activity, as reported by Dhaouafi et al. (2024) for protein hydrolysates (MW 300–1800 Da) from *Gelidium spinosum* (red algae) [130].

A potent ACE inhibitory activity has also been reported by McLaughlin (2021) for protein hydrolysates from Palmaria palmata (red algae), Kumagai et al. (2020) for protein sequences from Mazzaella japonica (red algae), and Cermeño et al. (2019) for peptide sequences TYIA and YLVA from Porphyra dioica (red algae) [286,287,288]. The antihypertensive effects of peptides from macroalgae have also been noted in studies by Feng et al. (2021) on *Undaria pinnatifida* (brown algae), Zheng et al. (2020) on *Sargassum mcclurei* (brown algae), and Sun et al. (2019) on *Ulva intestinalis* (green algae) [133,144,289]. Additionally, pigments exhibit ACE inhibitory activity; for instance, Raji et al. (2020) demonstrated that fucoidans from *Sargassum wightii* (brown algae) possess this property [290].

**Table 10:** Biocompounds of marine algae with biological activity results in antimicrobial, anti-inflammatory diseases, cardioprotective, and ACE inhibitory activity.

Type of Seaweed	Bioactive Metabolites/Compounds	Mechanism of Action	Biological Activity	References
**Antimicrobial activity**				
Terpenoides				
*Dictyota dichotoma*—brown algae	Diterpenes as dictyols.	Have effects on cell viability in murine macrophage cell line RAW 264.7	Antimicrobial potential	[114]
*Kappapycus alvarezii*—red algae	Purified terpenoids fractions	Minimum inhibitory concentration (MIC) value was 1.5 mg/mL against *S. mutans.* Minimal bactericidal concentration (MBC) value was 3.0 mg/mL	Antimicrobial activity	[263]
*Gracillaria dura*—red algae	hexadecanoic acid methyl ester, n-hexadecenoic acid, 11-octadecanoic acid, and phytol	Minimum inhibitory concentration (MIC) value was 0.065 mg/mL against *S. mutans.* Minimal bactericidal concentration (MBC) value was 0.12 mg/mL	Antimicrobial activity	[263]
*Ulva lactuca*—green algae	Sesquiterpenoid as neophytadiene	Showed excellent inhibitory effects with the maximum activity (by diffusion) against *E. coli*, *K. pneumonia*, and *S. typhi*	Antimicrobial activity	[267]
*Stypopodium zonale*—brown algae	Atomaric acid and 4-acetoxydolastane, secundary metabolites,	Anti-HSV-2 activity with low cytotoxicity, inactivated 90% of the viral particle.	Anti-Herpes simplex virus	[268]
*Canistrocarpus cervicornis*—brown algae	Atomaric acid and 4-acetoxydolastane	Anti-HSV-2 activity with low cytotoxicity	Anti-Herpes simplex virus	[269]
*Canistrocarpus cervicornis*—brown algae	Dolastane-type diterpenoids	For *Chikungunya virus*, the compound was able to inhibit around 90% of the virus infectivity and for *Zika* virus, the effects were at approximately 64%	Anti-viral activity on *Zika* and *Chikungunya* viruses	[269]
Pigments				
*Sargassum angustifolium*—brown algae	Fucoxanthin	Showed antimicrobial activity against *S. aureus* by diffusion method	Antimicrobial activity	[270]
*Gongolaria indica*—brown algae	Fucoxanthin	Significant inhibition zone against *E. Coli* and *S. aureus*.	Antimicrobial activity	[270]
Polyphenols				
*Padina pavonica*—brown algae	Protocatechuic acid; *p*-*h*droxybenzoic acid; *p*-coumaric acid; *t*-ferulic acid; *o*-coumaric acid	Inhibition zone: *B. subtilis*: 12.7 ± 0.6 mm; *P. aeruginosa*: 15.7 ± 2.1 mm; *S. aureus*: 10.3 ± 1.5 mm and *C. albicans*: 10 ± 0.9 mm	Antimicrobial activity	[254]
**Anti-inflammatory activity**				
Polysaccharides				
*Sargassum autumnale*—brown algae	Fucoidan	Down-regulation of iNOS and COX2 and signaling pathways (NF-κB and MAPK).	Anti-inflammatory activity	[271]
*Sargassum siliquastrum*—brown algae	Fucoidan	Down-regulated the expression of inflammatory mediators (NO, PGE2, iNOS, COX-2), and pro-inflammatory cytokines via regulating MAPK and NF-κB.	Anti-inflammatory activity	[272]
*Sargassum confusum*—brown algae	Fucoidan	Reducing the expression of inflammatory mediators through regulation of NF-κB and MAPKs signaling pathways via activating Nrf2/HO-1 signaling.	Anti-inflammatory activity	[273]
*Cystoseira crinita*—brown algae	Fucoidan	Decreases IL-1β production.	Anti-inflammatory activity	[274]
*Sargassum swartzii*—brown algae	Sulfated Polysaccharide (fucoidan)	Inhibition of inflammatory mediators and pro-inflammatory cytokines.	Anti-inflammatory activity	[275]
*Codium fragile*—green algae	Sulfated polysaccharides	Decreases cell death and the generation of NO and ROS.	Anti-inflammatory activity	[276]
*Saccharina japonica*—brown algae	Sulfated galactofucan	Decreases cell death and the generation of NO and ROS.	Anti-inflammatory activity	[277]
*Sargassum binderi*—brown algae	Sulfated polysaccharides	Decreases LPS-induced cell death and NO production.	Anti-inflammatory activity	[278]
*Sargassum fulvellum*—brown algae	Sulfated polysaccharides	Decreases cell death and the generation of NO and ROS.	Anti-inflammatory activity	[279]
*Saccharina japonica*—brown algae	Sulfated polysaccharides	Decreases cell death and the generation of NO and ROS.	Anti-inflammatory activity	[280]
Fatty acids				
*Fucus spiralis*—brown algae	EPA and octadecatetraenoic acid	Showed dose-dependent effect on murine macrophage RAW 264.7 cell line,	Anti-inflammatory activity	[120]
*Undaria pinnatifida*—brown algae	SA, EPA	SA: IC50 values of 160 µg on ear for edema, 314 µg on ear for erythema, 235 µg on ear for blood flow. EPA: IC50 values of 230 µg on ear for edema, 462 µg on ear for erythema, 236 µg on ear for blood flow.	Anti-inflammatory activity	[120]
*Palmaria palmata*—red algae	Palmitic acid, Oleic acid, and EPA	Inhibition of the COX-2 enzyme	Anti-inflammatory activity	[117]
*Gracilaria gracilis*—red algae	SFA, MUFA, PUFA, HUFA, omega-3, omega-6	Inhibition of the COX-2 enzyme	Anti-inflammatory activity	[118]
*Undaria pinnatifida*—brown algae	SFA, MUFA, PUFA, HUFA, omega-3, omega-6	Inhibition of the COX-2 enzyme	Anti-inflammatory activity	[118]
*Ulva lactuca*—green algae	SFA, MUFA, PUFA, omega-3, omega-6	Inhibition of the COX-2 enzyme	Anti-inflammatory activity	[281]
*Ulva intestinalis*—green algae	SFA, MUFA, and PUFA	Inhibition of the COX-2 enzyme	Anti-inflammatory activity	[282]
Pigments				
*Sargassum fusiformis*—brown algae	Fucoxanthin, pheophytin-α, chlorophyll-a, β-carotene	Inhibiting production of prostaglandin E2 (PGE2), cyclooxygenase-2, interleukin (IL)-1β, and IL-6 from exposed HaCaT keratinocytes	Anti-inflammatory activity	[283]
**Cardioprotective activity and** **ACE inhibitory activity**				
Polysaccharides				
*Sargassum wightii*—brown algae	Sulfated polygalacto-pyranosyl-fucopyranan	The formation of hydrogen bonds with Zn^2+^ and other amino acid residues by the electronegative functionalities can lead to effective inhibition of ACE.	Antihipertensive activity	[235]
*Fucus vesiculosus*—brown algae	Fucoidan	Decreases lipid levels and the carotid atherosclerotic plaque formation.	Cardioprotective activity	[284]
Fatty acids				
*Alaria esculenta*—brown algae	Palmitic acid, Oleic acid and EPA	Showed high content of linoleic acid which indicates potential activity on coronary heart disease	Coronary hearth diseases,	[117]
*Palmaria palmata*—red algae	Palmitic acid, Oleic acid and EPA	Inhibition of the COX-2 enzyme	Coronary hearth disease	[117]
*Undaria pinnatifida*—brown algae	SFA, MUFA, PUFA, HUFA, omega-3, omega-6	Inhibition of the COX-2 enzyme	Cardioprotective activity	[118]
*Gracilaria gracilis*—red algae	SFA, MUFA, PUFA, HUFA	Inhibition of the COX-2 enzyme	Cardioprotective activity	[118]
*Ulva lactuca*—green algae	SFA, MUFA, PUFA	Inhibition of the COX-2 enzyme	Cardioprotective activity	[281]
*Ulva intestinalis*—green algae	SFA, MUFA, and PUFA	Inhibition of the COX-2 enzyme	Cardioprotective activity	[282]
*Curdiea racovitzae*—red algae	SFA, MUFA, PUFA	Inhibition of the COX-2 enzyme	Cardioprotective activity	[282]
Proteins				
*Sphaerococcus coronopifolius*—red algae	Protein hydrolysates with MW 300–1800 Da	ACE inhibitory activity IC50 = 160.32 µM; ACE inhibitory activity IC50 = 656.15 µM;	ACE inhibitory activity	[130]
*Gelidium spinosum*—red algae	Protein hydrolysates with MW 300–1800 Da (Fractions F1–F10)	F4: ACE inhibitory activity IC50 = 149.35 µM; F6: ACE inhibitory activity IC50 = 656.15 µM;	ACE inhibitory activity	[130]
*Palmaria palmata*—red algae	Protein hydrolysates	Positive role in glucose transport, increasing glucose uptake	ACE inhibitory activity	[286]
*Mazzaella japonica*—red algae	Protein sequences	Significant IC50 values were found in sequence IY from the peptide chain	ACE inhibitory activity	[287]
*Porphyra dioica*—red algae	Peptides sequences	TYIA: ACE inhibitory activity and YLVA: DPP-IV inhibitory activity	ACE inhibitory activity	[288]
*Undaria pinnatifida*—brown algae	Peptide	ACE inhibitory activity with IC50 = 225.87 μM	Antihypertensive activity	[133]
*Sargassum mcclurei*—brown algae	Peptide sequence	activity on endothelin-1 suppressing capacity for ACE inhibitory activity	Antihypertensive activity	[144]
*Ulva intestinalis*—green algae	Peptide sequences	Sequences: FGMPLDR: ACE inhibitory and MELVLR: ACE inhibitory	Antihypertensive activity	[289]
Pigments				
*Sargassum wightii*—brown algae	Fucoxanthin	Showed inhibition of ACE with half maximal inhibitory value	ACE inhibitory activity	[290]

### 4.6. Antidiabetic Activity

Table 11 presents bioactive compounds from marine algae with antidiabetic, anticoagulant, and metabolic disease properties, along with other health benefits. Ahmed et al. (2024) highlighted the antidiabetic potential of marine macroalgae, proposing two mechanisms for diabetes management: lowering blood glucose levels and reducing diabetic complications [212]. They identified several compounds with antidiabetic effects, including polysaccharides (fucoidans, alginates, and laminarins), carotenoids (fucoxanthin), phlorotannins, and sterols (fucosterols) found in marine algae [212]. Further studies support these findings. Lin et al. (2023) demonstrated the antidiabetic activity of fucoidan from *Sargassum pallidum* (brown algae) [291]. Thambi et al. (2022) reported that sulfated pyruvylated polysaccharide from *Gracilaria edulis* (formerly *Hydropuntia edulis*) (red algae) exhibits an anti-hyperglycemic effect [292]. Maneesh et al. (2018) found that sulfated polygalacto-pyranosyl-fucopyranan compounds from *Sargassum wightii* (brown algae) show significant antidiabetic potential for type 2 diabetes treatment [235].

### 4.7. Activities in the Treatment of Metabolic Diseases

Metabolic diseases are often challenging to detect and manage. Table 11 summarizes studies that explore the use of biocompounds from marine macroalgae—specifically fatty acids and minerals—as alternative treatments for these conditions. Research has highlighted the beneficial effects of fatty acids derived from marine algae. Rocha et al. (2021) reported that fatty acids from *Undaria pinnatifida*, a type of brown algae, exhibit positive effects [118]. Similarly, Berneira et al. (2020) demonstrated that fatty acids, including SFA, MUFA, and PUFA, extracted from *Ulva intestinalis* (green algae) and *Curdia racovitzae* (red algae) have beneficial impacts on metabolic diseases [282]. Minerals also play a crucial role in metabolic processes. Xavier et al. (2020) reviewed studies indicating that iron and zinc are essential for human metabolic activities [293]. Iron is particularly vital for physiological functions, as it is required for the production of hemoglobin and myoglobin, which facilitate oxygen transport throughout the body. Zinc contributes to metabolism, immune function, and cellular repair. According to Xavier et al. (2020), *Valoniopsis pachynema* (green algae) contains the highest levels of iron, while *Gelidium latifolium* (red algae) has the highest concentration of zinc [293].

### 4.8. Anticoagulant Activity

Polysaccharides from all types of macroalgae exhibit anticoagulant properties described in various studies, as shown in Table 11. Mendes Marques et al. (2019) reported anticoagulant activities for polysaccharide extracts from *Udotea flabellum*—green algae [223]. de Carvalho et al. (2020) reported anticoagulant activities of polysaccharide extracts from the green algae *Ulva lactuca* (formerly *Ulva fasciata*), Chagas et al. (2020) for polysaccharide extracts from *Gelidiella acerosa*—red algae—and Sun et al. (2018) for polysaccharide extracts from *Sargassum fusiforme* [294,295,296]. The anticoagulant activity is supported by polysaccharide biocompounds (polycarboxyl ulvans, sulfated polysaccharides, sulfated galactan, and polysaccharides with low MW) [296].

### 4.9. Neuroprotective Activity and Alzheimer’s Disease (AD)

The classes of biocompounds that have shown beneficial effects in neuroprotective conditions and in the treatment of Alzheimer’s disease are pigments, polysaccharides, and fatty acids, as shown in Table 11. Cho et al. (2018) evidenced that carotenoid pigments from seaweed have multiple activities such as antioxidant, anti-inflammatory, and autophagy-modulating activities in the context of neurodegenerative diseases [297].

The mechanisms of treatment of Alzheimer’s disease with seaweed extracts (poly/oligosaccharides) include the following: (I) anti-inflammatory and antioxidant activities; (II) scavenging of free radicals; (III) inhibition of ROS production and inhibition of nitric oxide (NO) and prostaglandin formation; (IV) decreased expression of mitochondria-mediated proteins and protein aggregation; (V) direct interaction with the aggregated peptide, preventing Aβ oligomerization and fibrillation; (VI) attenuation of Aβ-induced apoptosis through the JNK pathway; and (VII) impact on gut microbial processing and subsequent neuroinflammation. Bauer et al. (2021) highlighted the use of algal polysaccharides for the treatment of neurodegenerative diseases such as Alzheimer’s disease [298]. Park et al. found that mice treated with fucoidan extracts from *Ecklonia cava*—brown algae—experienced beneficial effects in the treatment of Alzheimer’s disease [299]. Bogie et al. (2019) showed that the use of fatty acid extracts from *Sargassum fusiforme* in the treatment of Alzheimer’s disease led to beneficial effects [300].

### 4.10. Antiprotozoal Activity

The effects that were generated by the appearance of malaria, leishmanicide, and trypanocide have required multiple studies to detect natural biocompounds that are effective in antiprotozoal activities. In this sense, Hassan et al. (2021) studied phenolic compounds from *Padina boryana*—brown algae—and showed that they exhibit antiprotozoal activity against *Trypzanosoma cruzi*, and against *Leishmania donovani*, as shown in Table 11 [255].

### 4.11. Bone Deficiencies

This condition is quite widespread both in children and especially in the elderly. Xavier et al. (2020) show that in the treatment of bone deficiencies, Ca intake is important, as shown in Table 11 [293]. Calcium obtained from seaweed helps increase bone density regardless of age. The authors show that the disadvantage of calcium obtained conventionally through calcium supplements can also generate other gastrointestinal side effects, such as bloating, nausea, and constipation [293]. It has been indicated that algal calcium also possesses other essential minerals (magnesium, phosphorus, potassium, and zinc) and vitamins such as vitamin C, D [293]. Mohan et al. (2023) showed that seaweed has applications in the treatment of bone deficiencies through the content of minerals, vitamins, and proteins that they possess [301].

### 4.12. Malnutrition

Folic acid and vitamin B12 deficiency is common in children, along with iron deficiencies. Koseki et al. (2023) showed that vitamin B12 (Cyanocobolamin) and B9 (folic acid) deficiency disrupts the biosynthesis of methionine necessary for the accumulation of homocysteine, which is a risk factor for many diseases, as shown in Table 11. The authors demonstrated that these deficiencies can be remedied by supplementation with vitamin B9 and Vitamin B12 from marine macroalgae [302].

## 5. Nutraceutical Applications

Functional food or nutraceutical food are foods that provide not only nutritional value but can also help prevent health problems. Seaweed-based foods are considered nutraceutical products due to their positive effects on human health, for example, in anti-cancer diseases, in cardiovascular diseases for diabetes amelioration and as antioxidants, antimicrobials, and anti-inflammatory agents, as reported by Lomartire et al. (2021) [19].

### 5.1. Anti-Nutritional Compounds

Several studies have identified the presence of anti-nutritional compounds in seaweeds. Ahmed et al. (2024) reported that seaweeds contain anti-nutrients such as phytic acid, polyphenolic compounds (e.g., tannins), phlorotannins, lectins, and inhibitors of amylase and trypsin, which can impact the bioavailability and digestibility of nutrients like proteins and trace elements [212]. However, Choudhary et al. (2023) noted that these compounds were found in such small quantities that they are considered harmless. Their study also highlighted that while alkaloids can be toxic at high concentrations due to their interference with electrochemical transmissions in the nervous system, they are safe at low levels [53]. Additionally, Choudhary et al. (2023) detected only trace amounts of tannic acid and phytic acid in various seaweed samples, making their presence negligible, and found no detectable saponins, which can affect metabolism by inhibiting certain digestive enzymes [53].

Beyond anti-nutritional compounds, Xu et al. (2023) warned that nutrient losses in seaweed-derived products can occur due to processing and poor digestion in the human body [213]. Another concern, highlighted by Ownsworth et al. (2019), is seaweeds’ tendency to accumulate metals, and other pollutants [303]. Studies have shown that the accumulation of inorganic arsenic increases the risk of cancer, nervous system disorders, and cardiovascular diseases [213,303]. Cadar et al. (2018, 2019) studied the content of potentially toxic heavy metals (Pb, Cd, Cu, and Zn) included *in Cystoseira barbata*, *Ceramium rubrum*, *Ulva lactuca*, *Ulva intestinalis*, and *Cladophora vagabunda*, harvested from the Black Sea, and showed the maintenance of low levels of pollution of contaminants of algal biomass in terms of heavy metal content [9,304].

Given these concerns, Ahmed et al. (2024), Xu et al. (2023), and Silva et al. (2024) emphasized the need to develop high-value seaweed-based foods while detecting and minimizing toxic substance concentrations to ensure they meet health standards [212,213,214].

### 5.2. Nutraceutical Applications of Marine Algae Products

Based on the medical benefits of biocompounds from marine macroalgae, these algae can be used as functional foods in various forms, such as staple foods or beverages. Mendes et al. (2022) highlight that algae-based foods have become increasingly important in Europe, as they serve as a natural source of micro- and macronutrients, along with trace elements, which enhance their nutritional and pharmacological value [305]. Peñalver et al. (2020) argue that algae are a natural resource of significant interest, as they contain compounds with diverse biological activities and can be used as functional ingredients in numerous technological applications to create functional foods [116]. For example, plant-based oils contain other bioactive compounds, such as oil-soluble vitamins, phytosterols, tocopherols, and pigments, but terrestrial crops typically lack essential ω-3 fatty acids, like EPA or DHA. Belkacemi et al. (2020) note that seaweed and phytoplankton are the primary producers of ω-3 polyunsaturated fatty acids (PUFAs), making them a promising source for nutraceutical applications [306]. Geranpour et al. (2020) reported studies on the spray-drying encapsulation of fatty acids and functional oils [307]. Mouritsen et al. (2019) demonstrate that seaweed can be consumed as food or as an ingredient in prepared foods in various forms, including fresh, fermented, dried, or frozen, either whole or processed into flakes, granules, or powders [308]. Cornish et al. (2019) describe how, in Brittany, dulse and kombu are used to prepare the traditional “bread of the sea” (bara mor), while minced seaweed in butter (beurre des algues) is used to cook fish or spread on bread to accompany shellfish [309]. Granato et al. (2020) note that the current trend is to select new natural resources with added health benefits, specifically functional foods [310]. Tanna et al. (2018) emphasize the growing interest in secondary metabolites from marine macroalgae with antioxidant properties, particularly for nutraceutical applications [311]. El-Beltagi et al. (2022) provide a comprehensive review that characterizes active biocompounds as secondary metabolites, helping to understand the progress and limitations of seaweeds’ bioactivity as nutraceuticals [312]. Cotas et al. (2020) mention that several macroalgae products possess exceptional nutraceutical, pharmacological, and biomedical properties, with key compounds including fatty acids, pigments, phenols, and polysaccharides [313]. Ganesan et al. (2019) demonstrate that seaweed bioactives, including polysaccharides, pigments, fatty acids, polyphenols, and peptides, exhibit various beneficial biological properties that could enhance functional foods and nutraceutical products [314]. Din et al. (2022) show that brown algae can be used as a functional food source of fucoxanthin [3]. André et al. (2021) indicate that brown algae can be used as a functional food to combat hypercholesterolemia [15]. Barot et al. (2019) suggest that the nutritional composition of seaweeds makes them a potential source of natural food [314]. Lastly, Shannon et al. (2019) discuss recent advancements in the use of seaweed for human health from an epidemiological standpoint and as a functional food ingredient [315].

## 6. Conclusions

The active biocompounds in seaweeds make them, as a natural resource, attract attention not only as food but also as sources for various naturally pharmacologically active products and nutraceuticals. We can state that the medical and nutraceutical benefits of seaweeds are supported by the biological activities documented on multiple studies related to antitumor, antioxidant, cardioprotective, antimicrobial, and anti-inflammatory effects, as well as their role in the management of metabolic diseases, malnutrition, and Alzheimer’s disease. This review is important because it primarily utilizes a systematic and unitary approach to the information on the classification and description of macroalgal species, their nutritional composition, and various extraction methods. Each category of biocompounds from the three algal types is analyzed in terms of chemical structure, extraction techniques, and quantitative analysis. The characterization of algal biocompounds, as active secondary metabolites, was also complemented by their medical and nutraceutical applications, in alternative treatments for various ailments compared to classical medicine that uses drugs obtained by chemical synthesis.

A significant challenge, however, is the potential presence of harmful pollutants (e.g., heavy metals, high iodine content, or other toxic compounds), which need to be carefully considered in terms of risks to human health. The growing interest in bioactive substances with proven therapeutic potential has led to the need for modern, environmentally friendly extraction, purification, and analysis techniques. These advances are essential for the development of value-added functional products that promote human health.

Future directions for research on seaweed-derived bioactive compounds need to focus on the optimization of modern extraction techniques (such as enzyme-assisted and ultrasound-assisted methods) to improve the efficiency and yield of extractions. The discovery of novel compounds from unexplored seaweed species presents opportunities for pharmaceutical and nutraceutical applications. More in-depth clinical studies are also needed to validate their health benefits, bioavailability, and safety.

Exploring the incorporation of algae as functional foods and supplements could enhance both their commercial viability and contribution to global food security. Advances in marine biotechnology, including genetic engineering, may boost the production of high-value compounds, while sustainable farming methods are crucial for meeting rising demand without harming marine biodiversity. Further research should investigate their potential in preventing chronic diseases such as cancer, cardiovascular disease, diabetes, and neurodegenerative disorders. Additionally, nanoformulation strategies could improve the stability and delivery of bioactive compounds, opening new avenues for targeted drug therapies.

With rising inflation in recent years, the commercial use of algae for health and nutrition shows promising growth. However, making seaweed more palatable remains a key challenge for consumer acceptance as a functional food. Meeting the demand for raw seaweed requires both cost-effective production and the exploration of previously overlooked natural sources.

## Figures and Tables

**Table 1:** Composition data on proximate nutritional composition from macroalgae (results are expressed in mean % ± standard deviation).

	**Green algae**											
	***Ulva lactuca***	***Ulva lactuca***	***Ulva rigida***	***Ulva lactuca* (as *Ulva fasciata*)**	***Cladophora vagabunda***	***Cladophora vagabunda***	***Acrosiphonia orientalis***	***Caulerpa scalpelliformis***	***Caulerpa lentillifera***	***Ulva intestinalis* (as *Enteromorpha intestinalis*)**	***Ulva flexuosa*** **(as *Enteromorpha flexuosa*)**	***Ulva intestinalis* (as *Enteromorpha intestinalis*)**
	Black Sea	Arabian Sea	Atlantic waters	Indian waters	Black Sea	Black Sea	Arabian Sea	Arabian Sea	Atlantic waters	Black Sea	Indian waters	Gulf Gökova Aegean Sea
Moisture (%)	10.85 ± 0.26	25.0 ± 1.0	-	-	5.71 ± 0.92	11.98 ± 0.84	19 ± 2.0	20.0 ± 1.0	-	11.98 ± 0.84	-	12.14 ± 1.11%
Ash (%)	23.62 ± 0.59	16.0 ± 2.0	28.6	27.0 ± 0.024	26.38 ± 0.31	24.63 ± 0.84	22 ± 2.0	15.0 ± 2.0	24–37	25.65 ± 0.98	32.2 ± 0.92	14.81 ± 0.23
Sulphates (%)	70.46 ± 1.87	-	-	-	67.92 ± 0.53	68.65 ± 1.78	-	-	-	67.68 ± 1.63	-	-
Nitrogen (%)	2.26 ± 0.48	66.0 ± 4.0	-	-	2.45 ± 0.02	2.39 ± 0.26	41 ± 3.0	51.0 ± 2.0	-	2.18 ± 0.39	-	-
Protein (%)	14.13 ± 0.85	6.0 ± 1.0	18–19	22.7 ± 0.22	15.43 ± 0.36	14.94 ± 0.92	7 ± 0.2	6.0 ± 1.0	10–13	13.63 ± 0.96	17.29 ± 1.24	13.42 ± 1.31
Lipid (%)	2.78 ± 0.69	1.0 ± 0.5	0.9–2.0	0.89 ± 0.12	3.85 ± 0.47	2.86 ± 0.75	3.0 ± 0.2	4.0 ± 0.5	0.86–1.11	1.72 ± 0.56	0.76 ± 0.24	1.31 ± 0.5
Carbohydrates (%)	58.36 ± 1.64	56.0 ± 2.0	43–56	32.0 ± 0.04	48.45 ± 0.5	62.37 ± 1.74	16 ± 2.0	23 ± 2.0	38–59	60.68 ± 1.36	30.1 ± 0.18	58.03 ± 2.31
Total dietary fiber (%)	60.56 ± 1.1	11.0 ± 0.9	38–41	-	61.56 ± 1.5	63.35 ± 1.24	27.0 ± 0.5	24.0 ± 2.2	33	59.66 ± 1.95	-	52.36 ± 3.26
References	[49]	[53]	[54]	[55]	[30]	[49]	[53]	[53]	[54]	[49]	[55]	[56]
	**Red algae**											
	***Halymenia porphyriformis***	***Palmaria palmata***	***Porphyra umbilicalis***	***Acanthophora spicifera***	***Gracilaria edulis***	***Ceramium virgatum* (as *Ceramium rubrum*)**	***Jania pedunculata* var. *adhaerens* (as *Jania adhaerens*)**	***Gracilaria corticata***	***Scinaia carnosa***	***Gracilaria edulis***	***Gracilaria corticata***	***Laurencia obtusa***
	Arabian Sea	Atlantic waters	Atlantic waters	Indian waters	Indian waters	Black Sea	Sri Lanka coastal area	Sri Lanka coastal area	Arabian Sea	Southeast coast of India	Southeast coast of India	Red sea coast
Moisture (%)	19.0 ± 1.1	-	-	-	-	11.01 ± 0.13	92.96 ± 0.27	96.32 ± 0.02	21.0 ± 2.0	10.40 ± 0.69	8.40 ± 0.65	-
Ash (%)	17.0 ± 1.2	12–37	12	21.0 ± 0.08	22.8 ± 0.04	13.83 ± 1.68	05.01 ± 0.01	07.15 ± 0.01	46.0 ± 2.0	7.36 ± 0.39	8.10 ± 0.49	-
Sulphates (%)	-	-	-	-	-	75.16 ± 1.56	-	-	-	-	-	-
Nitrogen (%)	75. 0 ± 8.0	-	-	-	-	3.19 ± 0.41	-	-	45.0 ± 8.0	-	-	-
Protein (%)	3.0 ± 1.0	8–35	29–39	20.2 ± 0.12	18.04 ± 0.03	19.94 ± 2.56	29.47 ± 0.15	28.70 ± 0.46	2.0 ± 0.1	25.29 ± 0.67	22.84 ± 0.87	5.41 ± 0.11
Lipid (%)	1.0 ± 0.4	0.7–3	0.3	0.48 ± 0.04	0.72 ± 0.04	3.43 ± 0.25	1.52 ± 0.08	1.66 ± 0.18	3.0 ± 1.0	4.76 ± 0.73	7.07 ± 0.33	3.04 ± 0.12
Carbohydrate (%)	22 ± 1.0	46–56	43.0	26.2 ± 0.02	24.8 ± 0.12	51.90 ± 4.35	-	-	30.0 ± 1.0	4.71 ± 0.60	8.30 ± 0.49	20.17 ± 0.1
Total dietary fiber (%)	3.0 ± 0.2	29–46	29–35	-	-	-	56.81 ± 0.38	59.15 ± 0.76	5.0 ± 0.5	-	-	-
References	[53]	[54]	[54]	[55]	[55]	[57]	[58]	[58]	[53]	[59]	[59]	[60]
	**Brown algae**											
	***Sargassum linearifolium***	***Fucus vesiculosus***	***Laminaria digitata***	***Undaria pinnatifida***	***Saccharina latissima***	***Padina gymnospora***	***Gongolaria barbata* (as *Cystoseira barbata*)**	***Sargassum ilicifolium***	***Sargassum polycystum***	***Sargassum oligocystum***	***Himanthalia elongata***	***Sargassum asperifolium***
	Arabian Sea	Atlantic waters	Atlantic waters	Atlantic warers	Atlantic waters	Indian waters	Black Sea	Sri Lanka coastal area	Sri Lanka coastal area	Indo-West Pacific area	North-eastern Atlantic coast	Red Sea at Hurghada Coast
Moisture (%)	14.0 ± 1.0	-	-	-	-	-	9.27 ± 0.42	95.92 ± 0.37	92.58 ± 0.32	-	-	88.08
Ash (%)	24 ± 2.0	14–30	38	26–40	34.78	23.2 ± 0.03	17.63 ± 1.73	13.15 ± 0.41	18.48 ± 0.21	21.91 ± 0.28	-	19.60
Sulphates (%)	-	-	-	-	-	-	-	-	-	-	-	-
Nitrogen (%)	56 ± 3.0	-	-	-	-	-	-	-	-	-	-	-
Protein (%)	5 ± 1.0	3–14	8.15	12–23	6–6.26	12.07 ± 0.78	14.13 ± 2.11	28.02 ± 0.68	16.15 ± 0.33	9.26 ± 0.16	5.4 ± 0.46	3.50
Lipid (%)	7 ± 0.5	1.9	1.9	1.05–45	0.5–1.1	1.4 ± 0.82	1.03 ± 0.54	4.45 ± 0.12	4.50 ± 0.21	3.51 ± 0.21	17.06 ± 1.50	0.17
Carbohydrate (%)	53.0 ± 3.0	46.8	48	45–51	52–61	28.0 ± 0.12	58.05 ± 0.72	-	-	52.06	26.3	39.25
Total dietary fiber (%)	12 ± 0.5	43–59	37	16–51	30	-	-	51.46 ± 0.53	54.49 ± 0.95	-	53.3 ± 3.5	-
References	[53]	[54]	[54]	[54]	[54]	[55]	[57]	[58]	[58]	[61]	[62]	[63]

**Table 2:** Extraction methods and yields for different types of polysaccharides.

Polysaccharides	Species	Yield	Method	References
Carrageenans	*Kappaphycopsis cottonii* (formerly *Eucheuma cottonii*)—red algae	67.86%	Bead mill extraction	[91]
	*Chondracantus canaliculatus*—red algae	45.05%	UAE—Ultrasound-assisted extraction	[92]
	*Euchema cottonii*—red algae	30.20%	Conventional extraction method of semi-refined carrageenan	[93]
	*Gelidium corneum* (formerly *Gelidium sesquipedale*)—red algae	8.4%	Solubilization in hot water and alkali treatment	[94]
Agars	*Gelidium corneum* (formerly *Gelidium sesquipedale*)—red algae	10–12%	Sonication with hot water treatment	[95]
	*Gracilariopsis lemaneiformis*—red algae	12.32%	Alkaline extraction	[96]
	*Gracilariopsis lemaneiformis*—red algae	2.52%	EAE—Enzyme extraction	[96]
	*Gracilariopsis lemaneiformis*—red algae	5.33%	EA—Enzyme-assisted extraction	[96]
	*Nizamuddinia zanardinii*—brown algae	3.51%	UA—Ultrasound-assisted extraction	[97]
	*Fucus distichus* subsp. *evanescens* (formerly *Fucus evanescens*)—brown algae	4.44%	UAE—Ultrasound-assisted extraction	[98]
Fucoidans	*Nizamuddinia zanardinii*—brown algae	Alcalase, cellulase, flavourzyme, viscozyme, hot water 10–15%	EAE—Enzyme-assisted extraction	[99]
	*Sargassum fusiforme*—brown algae	11.24%	Conventional extraction with dilute hydrochloric acid	[100]
	*Sargassum wightii*—brown algae	14.61%	UAE—Ultrasound-assisted extraction	[101]
Alginates	*Ascophyllum nodosum*—brown algae	18.3–23.7%	UAE and Conventional method (HCl)	[102]
	*Turbinaria triquetra*—brown algae	22.2%	Conventional method (formaldehyde)	[103]
	*Gongolaria barbata*—brown algae	19%	Conventional method (HCl)	[104]
	*Ulva fenestrata*—green algae	9.03%	EAE–Enzyme assisted extraction	[105]
	*Ulva fenestrata*—green algae	8.65%	UAE–Ultrasound assisted extraction	[105]
Ulvans	*Ulva fenestrata*—green algae	17.92%	U-EAE—combined ultrasound with enzymatic extraction	[105]
	*Ulva lactuca*—green algae	36.4%	Conventional extraction (strong acid produces higher extraction yields)	[106]
	*Ulva prolifera*—green algae	36.38%	Microwave-assisted hydrothermal extraction	[107]
	*Ulva intestinalis*—green algae	12%	Conventional extraction (ethanol)	[108]

**Table 4:** Amino acids content from different green, red, and brown seaweeds (% percentage of the total amino acids).

	Green Algae		Red Algae					Brown Algae			
Type of Algae	*Ulva rigida*	*Ulva rigida*	*Palmaria palmata*	*Chondrus crispus*	*Porphyra dioica*	*Gracilaria gracilis*	*Gelidium corneum*	*Fucus spiralis*	*Ascophylum nodosum*	*Undaria pinnatifida*	*Sargassum mcclurei*
Proteins (% dw)	5.67	9.6	12.5	35.2	28.7	18.7	21	11.8	9.4	16.5	8.4
Essential amino acids (EAAs) (%)	-	40.8	37.7	40.9	39.8	45.6	44.1	38.7	39.2	37.2	27.8
Arginine (Arg)	0.7	6	6	6.5	2.3	1.4	-	1.5	1.7	2.7	3.8
Cysteine (Cys)	0.7	2.9	2.1	0.7	-	-	-	-	-	-	3.5
Glutamic acid (Glu)	1.4	-	15.5	12.1	3.1	12.5	1.6	7.2	7.2	7.6	29.7
Glycine (Gly)	1	6	5.8	5.2	1.8	1.3	0.8	-	-	0.2	4.2
Histidine (His)	0.2	-	4.6	2.1	0.6	0.1	0.3	1.6	1.1	1.4	1.3
Isoleucine (Ile)	0.2	4.4	3.6	4	-	0.9	0.9	1.9	1.6	2	3.7
Leucine (Leu)	0.7	7.8	5.9	6.9	2.2	1.2	1.6	-	2.3	3	6.2
Lysine (Lys)	0.5	4.7	5.6	5.3	2.2	1.3	1.2	3.7	3.3	2.8	4.1
Hydroxylysine (Hyl)	-	-	2.7	-	-	-	-	-	-	-	-
Methionine (Met)	0.2	1.3	-	3.3	0.5	0.3	0.1	0.2	0.4	0.7	1.3
Phenylalanine (Phe)	0.6	5.7	3.8	4.3	1.1	0.9	1	1.2	1.2	1.7	4
Proline (Pro)	0.6	4.4	4.4	5.6	0.9	0.9	1.5	-	-	-	3.9
Hydroxiproline (Hyp)	-	1	-	-	-	-	-	1.8	1.6	0.9	-
Threonine (Thr)	0.5	4.8	4.7	5.5	1.2	1	0.7	2.7	1.9	2.4	3.6
Valine (Val)	0.3	6.8	6.1	6.2	1.2	1	1.4	2.2	1.9	2.5	-
Alanine (Ala)	1	8.4	6.3	7.5	3	1.2	1.9	0.7	1.5	3.4	7.9
Aspartic acid (Asp)	2	12.5	10.2	12	3.3	2.1	2	5.2	4.1	4.3	8.2
Serine (Ser)	0.8	5.5	5	5.1	1.6	1.2	0.8	5.5	-	5.8	-
References	[140]	[141]	[138]	[138]	[141]	[142]	[143]	[139]	[139]	[139]	[144]

**Table 5:** Pigments content from diffent green, red, and brown marine macroalgae. Units of measurement are indicated in the first line of the table, at the top of each column.

**Typ of Algae**	**Region**	Total Clorophyll mg/g; mg/L *; µg/g **	**Clorophyll-*a*** mg/g; mg/L *; µg/g **	**Clorophyll-*b*** mg/g; μg/g **	Total Carotenoids mg/g; mg/L *; μg/g **	β-Carotene mg/g; g/100 g *; mg/100 g **; µg/g ***	Fucoxanthin mg/g; mg/100 g *; µg/g **	**Astaxanthin mg/100 g**	**Zeaxanthin mg/100 g;** µg/g *	Lutein mg/100 g; mg/g; *; µg/g**	**References**
**Green algae**											
*Ulva lactuca* (as *Ulva fasciata*)	Black Sea, Romanian coast	23.23 ± 0.67	19.16 ± 2.69	4.07 ± 0.36	9.97 ± 0.85	-	-	-	-	-	[30]
*Ulva lactuca* (as *Ulva fasciata*)	Black Sea, Romanian coast	35.37 ± 1.7	26.95 ± 1.5	8.42 ± 1.56	16.25 ± 1.3	-	-	-	-	-	[49]
*Ulva lactuca* (as *Ulva fasciata*)	Saurashtra Coast, India	14.00 ± 0.11 *	8.16 ± 2.69 *	4.97 ± 0.85	0.80 ± 0.02 *	-	-	-	-	-	[53]
*Ulva fasciata*	Indian waters	3.49 ± 0.62	2.09 ± 0.15	1.4 ± 0.46	0.60 ± 0.06	0.37 ± 0.02	-	-	-	-	[55]
*Ulva fasciata*	Coastal area of Philippines	6.82	2.18 ± 0.74	4.64 ± 0.6	-	0.72 ± 0.00	-	-	-	-	[161]
*Ulva intestinalis*	Black Sea, Romanian coast	20.97 ± 1.67	16.74 ± 1.65	4.25 ± 0.45	12.73 ± 1.32	-	-	-	-	-	[30]
*Ulva intestinalis*	Black Sea, Romanian coast	30.51 ± 1.82	23.56 ± 1.88	6.95 ± 1.6	15.98 ± 1.98	-	-	-	-	-	[49]
*Ulva flexuosa*	Indian waters	3.14 ± 0.09	1.90 ± 0.25	1.24 ± 0.06	0.49 ± 0.12	0.32 ± 0.04	-	-	-	-	[55]
*Cladophora vagabunda*	Black Sea, Romanian coast	43.67 ± 1.97	24.13 ± 2.57	19.54 ± 1.55	13.90 ± 0.42	-	-	-	-	-	[30]
*Cladophora vagabunda*	Black Sea, Romanian coast	41.64 ± 1.52	29.25 ± 1.56	12.39 ± 1.35	17.66 ± 1.56	-	-	-	-	-	[49]
*Acrosiphonia orientalis*	Saurashtra Coast, India	7.00 ± 0.05 *	-	-	11.00 ± 0.01 *	-	-	-	-	-	[53]
*Caulerpa scalpelliformis*	Saurashtra Coast, India	3.00 ± 0.05 *	-	-	8.00 ± 0.01 *	-	-	-	-	-	[53]
*Caulerpa racemosa*	Coastal area of Philippines	123.58	42.15 ± 0.21	81.42 ± 0.24	-	17.26 ± 1.88	-	-	-	-	[161]
*Caulerpa racemosa*	Indian waters	21.09 ± 0.60	10.14 ± 0.13	11.12 ± 0.57	11.45 ± 0.59	-	-	-	-	-	[162]
*Caulerpa racemosa*	Indonesian Coast	-	-	-	-	20.50 ± 0.10 *	1.40 ± 0.01 *	4.60 ± 0.10	4.70 ± 0.01	1.50 ± 0.50	[163]
*Caulerpa lentillifera*	Malaysian waters	7.29	3.32	3.97	63.47	10.7	-	-	21.30 *	21.13 **	[164]
*Chlorococcum infusionum* (as *Chlorococcum humicola*) (green microalga)	Thailand Coast	10.01 ± 0.13	5.90 ± 0.15	4.11 ± 0.03	2.01 ± 0.16	-	-	-	-	0.59 ± 0.12	[165]
**Red algae**											
*Scinaia carnosa*	Saurashtra Coast, India	1.50 ± 0.01 *	-	-	0.70 ± 0.01 *	-	-	-	-	-	[53]
*Halymenia porphyriformis*	Saurashtra Coast, India	7.00 ± 0.05 *	-	-	0.20 ± 0.01 *	-	-	-	-	-	[53]
*Gracilaria corticata*	Indian waters	-	-	-	-	4.13 ± 0.07 ***	6.06 ± 0.05 **	-	0.65 ± 0.04 *	0.26 ± 0.05 **	[166]
*Gracilaria corticata*	Southeast coast of India	-	8.96 ± 0.39 **	7.74 ± 0.33 **	12.82 ± 0.50 **	-	-	-	-	-	[59]
*Eucheuma denticulatum*	Malaysian waters	-	-	-	-	4.7 ± 0.1 **	4.0 ± 0.0 *	3.0 ± 0.0	21.3 ± 0.1	87.7 ± 0.1	[167]
*Gracilaria edulis*	Indian waters	0.79 ± 0.05	0.66 ± 0.26	0.13 ± 0.08	0.13 ± 0.02	0.11 ± 0.02	-	-	-	-	[55]
*Gracilaria edulis*	Southeast coast of India	-	17.14 ± 0.55 **	8.44 ± 0.63 **	2.99 ± 0.56 **	-	-	-	-	-	[59]
*Acanthophora spicifera*	Indian waters	1.41 ± 0.62	1.17 ± 0.18	0.24 ± 0.02	0.32 ± 0.12	0.24 ± 0.04	-	-	-	-	[55]
*Kappaphycus striatus*	Malaysian waters	4.52	3.41	1.1	57.02	7.59	-	-	4.4 *	38.6 **	[164]
*Gracilaria tikvahiae*	Malaysian waters	2.97	2.55	0.42	25.13	3.05	-	-	4.15 *	8.86 **	[164]
**Brown algae**											
*Laminaria saccharina*	Galician coastline from Spain	-	0.67	-	-	0.07	9.54	-	-	-	[16]
*Iyengaria stellata*	Saurashtra Coast, India	1.50 ± 0.02 *	-	-	0.7 ± 0.01*	-	-	-	-	-	[53]
*Sargassum linearifolium*	Saurashtra Coast, India	34.00 ± 0.27 *	-	-	3.0 ± 0.1 *	-	-	-	-	-	[53]
*Undaria pinnatifida*	Galician coastline from Spain	-	1.58	-	-	0.3	6.15	-	-	-	[168]
*Padina gymnospora*	Indian waters	2.13 ± 0.43	1.75 ± 0.42	0.38 ± 0.04	0.78 ± 0.08	0.48 ± 0.23					[55]
*Himanthalia elongate*	Atlantic North Coast	168.2 ± 15.0 **	67.6 ± 3.2 **	-	2.9 ± 0.3 **	-	2.79 ± 0.31 **	-	-	-	[153]
*Laminaria ochroleuca*	Atlantic North Coast	235.3 ± 15.4 **	183.5 ± 14.8 **	14.1 ± 0.5 **	27.0 ± 2.4 **	-	14.21 ± 0.31 **	-	-	-	[153]
*Undaria pinnatifida*	Atlantic North Coast	574.1 ± 33.2 **	321.3 ± 19.2 **	-	54.6 ± 1.3 **	-	26.81 ± 0.79 **	-	-	-	[153]
*Padina pavonica*	Malaysian water	7.51	3.4	-	100.89	9.14	-	-	10.87 *	7.21 **	[164]

**Table 6:** Phenols content from green, red, and brown seaweeds. Units of measurements are indicated in the first column of the table.

**Green Algae**													
**Algae**	***Ulva lactuca***	***Ulva lactuca***	***Ulva intestinalis***	***Ulva intestinalis***	***Ulva intestinalis***	***Ulva*** ***rigida***	***Chaetomorpha linum***	***Chaetomorpha* sp.**	***Halimeda macroloba***	***Cladophora vagabunda***	***Cladophora vagabunda***	***Caulerpa scalpelliformis***	***Acrosiphonia orientalis***
Region	Black Sea Romania	Arabian Sea	Black Sea	Black Sea Bulgaria	Western coast of Norway	Black Sea Bulgaria	Black Sea Coast Bulgaria	Arabian Gulf	Indonesian waters	Black Sea Romania	Black Sea Romania	Arabian Sea	Arabian Sea
TFC mg CE /100 g d.w. * mg QE/g d.w. **	15.6 ± 1.65 *	56 ± 9 **	13.1 ± 1.68 *	-	-	-	-	189.14 ± 0.99 **	-	-	12.3 ± 1.78 *	25.0 ± 5.0 **	277 ± 3.0 **
TPC mg GAE/100 g d.w. * mg GAE/g d.w. ** μgGAE/g d.w. ***	416.6 ± 1.56 *	285.5 ± 0.6 **	412.5 ± 1.26 *	512.8 ± 23.5 *	11.3 ± 1.4 **	32.80 ± 2.16 ***	403.9 ± 16.4 *	21.92 ± 0.43 **	186.80 ± 15.54 ***	356.8 ± 0.3 *	409.8 ± 1.68 *	26.0 ± 1.0 *	107 ± 1.0 **
References	[49]	[53]	[49]	[184]	[185]	[186]	[184]	[187]	[188]	[30]	[49]	[53]	[53]
**Red Algae**													
**Algae**	***Scinaia carnosa***	***Halymenia porphyriformis***	***Laurencia obtusa***	***Gracilaria* sp.**	***Hypnea pannosa***	***Jania rubens***	***Ellisolandia elongata***	***Gracilaria gracilis***	***Asparagopsis armata***	***Chondrus crispus***	***Gracilaria verrucosa***	***Gracilaria edulis***	***Eucheuma denticulatum***
Region	Arabian Sea	Arabian Sea	Red Sea Coast	Bali Coast	Saint Martin Island, Bangladesh	Egyptian waters	Egyptian waters	North coast of Tunisia	North coast of Tunisia	Red Sea Coast	Kupang, East Nusa Tenggara	Northwestern coast of Sri Lanka	Kenyan South Coast
TF C mgQE/g d.w. * mgCE/ g d.w. ** μg/g ***	95.0 ± 1.5 *	18.0 ± 1.0 *	4.78 ± 0.05 **	45.933 ± 0.56 *	43.12 ± 0.98 *	173.7 ± 6.8 *	69.7 ± 2.5 *	-	464 ± 0.63 **	202.66 ± 3.05 ***	-	541.02 ± 51.84 ***	9.36 ± 0.12 *
TPC mg. GAE/g d.w. * μgGAE/g d.w. **	31.0 ± 1.0 *	10.0 ± 1.0 *	7.83 ± 0.14 *	36.273 ± 0.2 *	89.89 ± 1.13 *	176.7 ± 6.9 *	22.9 ± 3.8 *	19.29 ± 1.8 *	14.95 ± 0.5 *	12.38 ± 2.31 **	11.27 *	1007.81 ± 54.21 **	146.15 ± 1.11 *
References	[53]	[53]	[60]	[189]	[190]	[191]	[191]	[192]	[192]	[193]	[194]	[195]	[196]
**Brown Algae**													
**Algae**	***Gongolaria barbata* (as *C. barbata*)**	***Iyengaria stellata***	***Sargassum linearifolium***	***Sargassum oligocystum***	***Himanthalia elongata***	***Sargassum asperifolium***	***Ericaria crinita***	***S. odonto-carpum* (as *S.coriifolium*)**	***Padina pavonica***	***Taoria atomaria***	***Phyllospora comosa***	***Ecklonia*** ***radiata***	***Cladostephus spongiosum***
Region	Black Sea	Arabian Sea	Arabian Sea	Indo-West Pacific Ocean area	North-eastern Atlantic Ocean	Red Sea at Hurghada Coast	Black Sea Coast Bulgaria	Saint Martin Island, Bangladesh	Egyptian waters	Egyptian waters	Australian Beach Coast	Australian Beach Coast	Mediterranean waters, Tunisia coast
TFC mg CE/g d.w. *	-	39 ± 4.0 *	200 ± 18.0 *	-	31.9 ± 2.65 *	-	-	58.29 ± 1.19 *	206.7 ± 4.7 *	374.1 ± 27.41 *	0.22 ± 0.01 *	0.03 ± 0.01 *	-
TPC mg. GAE/100 g d.w. * ppm **	358.6 ± 1.85 *	61 ± 1.0 *	61 ± 2.0 *	1.55 ± 0.11 *	52.7 ± 1.93 *	141.9 **	2662.4 ± 54.2 *	128.56 ± 0.59 *	152.5 ± 8.8 *	157.3 ± 5.9 *	3.01 ± 0.15 *	0.52 ± 0.05 *	10.91 *
References	[196]	[53]	[53]	[61]	[62]	[63]	[184]	[190]	[191]	[191]	[197]	[197]	[198]

**Table 8:** Mineral content from green, red, and brown seaweed. Units of measurements are indicated in the first column of the table.

	**Green algae**										
**Algae**	***Ulva lactuca***	***Ulva intestinalis***	***Cladophora vagabunda***	***Caulerpa scalpelliformis***	***Acrosiphonia orientalis***	***Ulva lactuca***	***Ulva rigida***	***Caulerpa lentillifera***	***Ulva lactuca***	***Ulva flexuosa***	***Ulva intestinalis***
Region	Black Sea	Black Sea	Black Sea	Arabian Sea	Arabian Sea	Arabian Sea	Atlantic waters	Atlantic waters	Indian waters	Indian waters	Gulf Gökova Aegean Sea
Minerals (Inorganic compounds)											
Na, mg/kg d.w.	825 ± 1.6	793.31 ± 1.20	853.15 ± 0.89	600 ± 110	1400 ± 125	2000 ± 100	1595	8917	20.12 ± 0.02	13.2 ± 0.8	-
K, mg/100 g d.w.	1120.54 ± 1.03	1230.56 ± 1.65	985.64 ± 2.03	9300 ± 250	4400 ± 120	3000 ± 220	1561	700–1142	27.2 ± 1.02	22.32 ± 1.08	1052.70
Ca, mg/100 g d.w.	1790.35 ± 2.55	1604.15 ± 2.96	1720.64 ± 2.87	44 ± 7.0	270 ± 30	62 ± 20	524	780–1874	740 ± 0.28	712 ± 0.04	15,977
Mg, mg/100 g d.w.	95.26 ± 1.05	90.87 ± 0.96	93.45 ± 0.91	800 ± 100	1400 ± 100	-	2094	630–1650	420 ± 0.02	436 ± 0.24	90.87 ± 0.96
Fe, mg/100 g d.w.	524.25 ± 0.64	490.36 ± 1.56	565.35 ± 1.05	0.5 ± 0.01	2.0 ± 0.01	0.40 ± 0.01	-	-	47 ± 0.04	40 ± 0.28	338.70
Zn, mg100 g d.w.; µg/100 g d.w. *	21.62 ± 0.65	24.74 ± 0.86	20.26 ± 0.85	2.0 ± 0.01	2.2 ± 0.01	4.00 ± 0.01	-	-	2.34 ± 0.48 *	1.518 ± 0.81 *	-
I (iodine content); mg/100 g); µg/100 g d.w. *	-	-	-	4.0 ± 1.0	15.0 ± 1.0	30 ± 11	-	-	38.89 ± 1.08 *	42.03 ± 1.02 *	-
References	[49]	[49]	[49]	[53]	[53]	[53]	[54]	[54]	[55]	[55]	[56]
	**Red algae**										
**Algae**	***Scinaia carnosa***	***Halymenia porphyriformis***	***Palmaria palmata***	***Porphyra umbilicalis***	***Acanthophora spicifera***	***Gracilaria edulis***	***Jania pedunculata***	***Gracilaria corticata***	***Gracilaria edulis***	***Gracilaria corticata***	***Laurencia obtusa***
Region	Arabian Sea	Arabian Sea	Atlantic waters	Atlantic waters	Indian waters	Indian waters	Sri Lanka coastal area	Sri Lanka coastal area	Southeast coast of India	Southeast coast of India	Red sea coast
Minerals (Inorganic compounds)											
Na; mg/100 g d.w.	1400 ± 70	2700 ± 30	1600–2500	940	36.08 ± 1.08	32.03 ± 0.28	86.23	67.06	-	-	102.55 ± 0.03
K; mg/100 g d.w.	25.2 ± 2.2	5800 ± 50.0	7000–9000	2030	52.08 ± 0.22	52.12 ± 0.07	121.61	125.82	-	-	870.38 ± 0.13
Ca; mg/100 g d.w.	70.0 ± 10.0	85.0 ± 15.0	560–1200	330	430 ± 0.14	410 ± 0.08	181.64	176.05	-	-	845.35 ± 0.11
Mg; mg/100 g d.w.	4000 ± 80	2200 ± 80	170–610	370	480 ± 1.02	580 ± 0.98	60.02	58.54	8.956 ± 0.77	46.32 ± 8.87	101.2 ± 0.13
Fe; mg/100 g d.w.	-	1.0 ± 0.01	-	-	52 ± 0.24	72 ± 0.24	73.97	49.48	55.736 ± 0.57	107.24 ± 20.9	-
Zn; mg/100 g d.w.; µg/100 g d.w. *	2.2 ± 0.01	3.00 ± 0.01	-	-	4.08 ± 0.28 *	5.21 ± 0.24 *	70.94	8.61	4.273 ± 2.12	3.152 ± 0.69	4.6 ± 0.05
I (iodin content); mg/100 g; µg/100 g d.w. *	2.0 ± 0.2	2.0 ± 0.1	-	-	64.8 ± 0.12 *	72.2 ± 0.08	-	-	-	-	-
References	[53]	[53]	[54]	[54]	[55]	[55]	[58]	[58]	[59]	[59]	[60]
	**Brown algae**										
**Algae**	***Iyengaria stellata***	***Sargassum linearifolium***	***Fucus vesiculosus***	***Laminaria digitata***	***Saccharina latissima***	***Padina gymnospora***	***Sargassum ilicifolium***	***Sargassum polycystem***	***Himanthalia elongata***	***Sargassum oligocystum***	***Sargassum asperifolium***
Region	Arabian Sea	Arabian Sea	Atlantic waters	Atlantic waters	Atlantic Waters	Indian waters	Sri Lanka coastal area	Sri Lanka coastal area	North-eastern Atlantic area	Indo-West Pacific area	Red Sea Coast
Minerals (Inorganic compounds)											
Na; mg/100 g d.w.	11,000 ± 250	7000 ± 130	2450–5469	3818	2620	36.36 ± 0.18	64.55	80.87	25.805 ± 7924	4.09 ± 0.13	-
K; mg/100 g d.w.	11,700 ± 400	6800 ± 190	2500–4322	11.5–79	4330	30.02 ± 0.17	127.47	127.66	57.480 ± 19.976	38.57 ± 8.57	12
Ca; mg/100 g d.w.	820 ± 35	300 ± 20	725–938	1005	810	820 ± 0.34	198.15	187.43	3469 ± 1526	30.95 ± 1.11	15.200
Mg; mg/100 g d.w.	1700 ± 110	900 ± 75	670–994	659	715	780 ± 0.08	84.73	86.38	3537 ± 1497	6.40 ± 0.13	5.778
Fe; mg/100 g d.w.	6.00 ± 0.12	0.30 ± 0.01	-	-	-	14.8 ± 0.32	48.50	128.46	17.8 ± 3.3	416.95 ± 4.24	0.802
Zn; mg/100 g d.w.; µg/100 g d.w. *;	2.60 ± 1.203	2.5 ± 1.1	-	-	-	4.19 ± 0.08 *	4.58	5.76	21.3 ± 13	21.84 ± 4.04	0.316
I (iodin content); mg100/g; µg/100 g d.w. *;	8.0 ± 1.0	41.0 ± 2.0	-	-	-	46.2 ± 1.03 *	-	-	**-**	3.79 ± 0.03	-
References	[53]	[53]	[54]	[54]	[54]	[55]	[58]	[58]	[61]	[60]	[63]

**Table 11:** Biocompounds of marine algae with biological activity results in antidiabetic activity, anticoagulant activity, metabolic diseases, and in other applications for health.

Type of Seaweed	Bioactive Metabolites/Compounds	Mechanism of Action	Biological Activity	References
**Antidiabetic activity**				
Polysaccharides				
*Sargassum pallidum*—brown algae	Fucoidan	Decreases lipid peroxidation. Reduces the activation of NF-κB signaling pathway.	Antidiabetic activity	[291]
*Gracilaria edulis*—red algae	Sulphated pyruvylated polysaccharide	Anti-hyperglycemic effect. Activities against type II transmembrane serine exopeptidase DPP-IV and carbolytic enzyme bundles.	Antidiabetic activity	[292]
*Sargassum wightii*—brown algae	Sulfated polysaccharide	Sulfated polygalacto-pyranosyl-fucopyranan could function as a potential pharmacophore lead against inflammation, type 2 diabetes.	Antidiabetic activity	[235]
**Metabolic Deseases**				
Fatty acids				
*Undaria pinnatifida*—brown algae	SFA, MUFA, PUFA, HUFA,	Inhibition of the COX-2 enzyme	Metabolic diseases	[118]
*Ulva intestinalis*—green algae	SFA, MUFA, and PUFA	Inhibition of the COX-2 enzyme	Metabolic diseases	[282]
*Curdiea racovitzae*—red algae	SFA, MUFA, PUFA	Inhibition of the COX-2 enzyme	Metabolic diseases	[282]
Minerals				
*Valoniopsis pachynema*—green algae	Iron	Iron is vital because it is used in the production of hemoglobin and myoglobin. The iron content was high in *V. pachynema.*	Metabolic activities	[293]
*Gelidium spinosum* (as *Gelidium latifolium*)*—red algae*	Zinc	Zinc is associated with metabolism and immune function. Zinc is involved in the repairing the body cells.	Metabolic activities	[293]
**Anticoagulant Activity**				
Polysaccharides				
*Udotea flabellum*—green algae	Sulfated galactan	Inhibited B16-F10 cell adhesion, migration, and proliferation.	Anticoagulant activity	[223]
*Ulva lactuca* (as *Ulva fasciata*)—green algae	Polycarboxyl ulvans	Anticoagulant activity by increasing the carboxyl groups.	Anticoagulant activity	[294]
*Gelidiella acerosa*—red algae	Sulfated polysaccharides	For anticoagulant, antiplatelet and antithrombotic activities, the mechanism of action is mainly due to the chemical structure of the isolated polysaccharide.	Anticoagulant, antiplatelet activity	[295]
*Sargassum fusiforme*—brown algae	Polysaccharides with low MW	These low MW polysaccharides possessed anticoagulant activity in the intrinsic, extrinsic, and common coagulation pathways.	Anticoagulant activity	[296]
**Neuroprotective Activity and Alzheimer’s Disease (AD)**				
Pigments				
Macroalgae	Carotenoids	The potent antioxidant properties of carotenoids may explain the neuroprotective effects of carotenoids by inhibiting neuroinflammation and activating autophagy.	Neuroprotective activity	[297]
Polysaccharides				
Macroalgae	Polysaccharides	Seaweed polysaccharides reduced lipid peroxidation and erythrocyte hemolysis.	Alzheimer’s disease	[298]
*Ecklonia cava*—brown algae	Fucoidan	Polysaccharide extracts inhibit BACE-1 protease, resulting in decreased amyloid-beta.	Alzheimer’s disease	[299]
Fatty acids				
*Sargassum fusiforme*	Fatty acids extract	Derived lipid extract to AD mice significantly improved short-term memory and reduced hippocampal Aβ plaque load by 81%	Alzheimer’s disease	[300]
**Antiprotozoal Activitaty**				
Polyphenolic compound				
*Padina boryana*—brown algae	Ellagic acid	Activity against *Trypzanosoma cruzi* showed a value of IC50 = 9.2 ± 0.87 µg/mL and against *Leishmania donovani*, showed IC50 = 8.87 ± 2.3 µg/mL	Antiprotozoal activity	[255]
**Bone Deficiencies**				
Minerals				
*Valoniopsis pachynema*—green algae	Calcium	Calcium to improve bone density	Bone deficiencies	[293]
*Ulva lactuca* (as *Ulva fasciata*)—green algae	Ca, Mg, Na, K, and Fe	Supplementing the body with exogenous daily intake. Calcium to improve bone density	Bone deficiencies	[301]
**Malnutrition**				
Vitamins				
*Saccharina japonica*—brown algae	Vitamin B9, B12	Supplementing daily intake	Malnutrition	[302]

## Abbreviations

SFE	Supercritical Fluid Extraction	HPLC	High-performance liquid chromatography
SWE	Subcritical Water Extraction	CG-MS	Gas Chromatography coupled with mass spectrometry
SFE	Supercritical Fluid Extraction	GP	Glycoproteins
PSE	Pressurized Solvents Extraction	AGPs	Arabinogalactan proteins
MAE	Microwave-Assisted Extraction	PBPs	Phycobiliproteins
EAE	Enzyme-Assisted Extraction	TPC	Total phenolic content
UAE	Ultrasound-assisted extraction	TFC	Total flavonoid content
CSE	Conventional Solvent Extraction	DPPH	2,2-diphenyl-1-picrylhydrazyl
SLE	Solid–Liquid Extraction	ABTS	2,2′-azino-bis(3-ethylbenzothi-azoline-6-sulfonic acid
LLE	Liquid–Liquid Extraction	TEAC	Trolox equivalent antioxidant capacity
CSE	Conventional solvent extraction	FRAP	Ferric reducing ability of plasm
MAPs	Marine algae Polysaccharides	AAS	Atomic absorption spectroscopy
AF	Fatty acids	ICP-MS	Inductively coupled plasma mass spectrometry.
MUFAs	Monounsaturated fatty acids	SFAs	Saturated fatty acids
PUFAs	Polyunsaturated fatty acids	EPA	Eicosapentaenoic acid
DHA	Docosahexaenoic acid	ACE inhibitors	Angiotensin-converting enzyme (ACE) inhibitors
